# mTORC1 links pathology in experimental models of Still’s disease and macrophage activation syndrome

**DOI:** 10.1038/s41467-022-34480-6

**Published:** 2022-11-28

**Authors:** Zhengping Huang, Xiaomeng You, Liang Chen, Yan Du, Kailey Brodeur, Hyuk Jee, Qiang Wang, Grace Linder, Roxane Darbousset, Pierre Cunin, Margaret H. Chang, Alexandra Wactor, Brian M. Wauford, Marc J. C. Todd, Kevin Wei, Ying Li, Anais Levescot, Yoichiro Iwakura, Virginia Pascual, Nicole E. Baldwin, Pierre Quartier, Tianwang Li, Maria T. Gianatasio, Robert P. Hasserjian, Lauren A. Henderson, David B. Sykes, Elizabeth D. Mellins, Scott W. Canna, Julia F. Charles, Peter A. Nigrovic, Pui Y. Lee

**Affiliations:** 1 Division of Immunology, Boston Children’s Hospital, Harvard Medical School, Boston, MA USA; 2 Division of Rheumatology, Inflammation, and Immunity, Brigham and Women’s Hospital, Harvard Medical School, Boston, MA USA; 3 Department of Rheumatology and Immunology, Guangdong Second Provincial General Hospital, Guangzhou, China; 4 Department of Orthopaedic Surgery, Brigham and Women’s Hospital, Harvard Medical School, Boston, MA USA; 5 Department of Rheumatology, The Second Affiliated Hospital of Zhejiang University School of Medicine, Hangzhou, China; 6 Blood Bank and Transfusion Medicine Division, Children’s Hospital of Philadelphia, Philadelphia, PA USA; 7 Université Paris Cité, Institut Imagine, INSERM UMR1163, Laboratory Intestinal Immunity, Paris, France; 8 Centre for Animal Disease Models, Research Institute for Biomedical Sciences, Tokyo University of Science, Chiba, Japan; 9 Department of Pediatrics and Drukier Institute for Children’s Health, Weill Cornell Medicine, New York, NY USA; 10 Baylor Scott & White Research Institute, Dallas, TX USA; 11 Pediatric Immunology, Hematology and Rheumatology Unit, Necker-Enfants Malades Hospital, Assistance Publique-Hopitaux de Paris, Universite de Paris, Paris, France; 12 Mass General Brigham Healthcare Center - Salem Hospital, Salem, MA USA; 13 Department of Pathology, Massachusetts General Hospital, Harvard Medical School, Boston, MA USA; 14 Center for Regenerative Medicine, Massachusetts General Hospital, Boston, USA; 15 Department of Pediatrics, Program in Immunology, Stanford University, Stanford, CA USA; 16 Division of Rheumatology, Children’s Hospital of Philadelphia, Philadelphia, PA USA

**Keywords:** Chronic inflammation, Paediatric research, Monocytes and macrophages, Inflammation

## Abstract

Still’s disease is a severe inflammatory syndrome characterized by fever, skin rash and arthritis affecting children and adults. Patients with Still’s disease may also develop macrophage activation syndrome, a potentially fatal complication of immune dysregulation resulting in cytokine storm. Here we show that mTORC1 (mechanistic target of rapamycin complex 1) underpins the pathology of Still’s disease and macrophage activation syndrome. Single-cell RNA sequencing in a murine model of Still’s disease shows preferential activation of mTORC1 in monocytes; both mTOR inhibition and monocyte depletion attenuate disease severity. Transcriptomic data from patients with Still’s disease suggest decreased expression of the mTORC1 inhibitors TSC1/TSC2 and an mTORC1 gene signature that strongly correlates with disease activity and treatment response. Unrestricted activation of mTORC1 by *Tsc2* deletion in mice is sufficient to trigger a Still’s disease-like syndrome, including both inflammatory arthritis and macrophage activation syndrome with hemophagocytosis, a cellular manifestation that is reproduced in human monocytes by CRISPR/Cas-mediated deletion of *TSC2*. Consistent with this observation, hemophagocytic histiocytes from patients with macrophage activation syndrome display prominent mTORC1 activity. Our study suggests a mechanistic link of mTORC1 to inflammation that connects the pathogenesis of Still’s disease and macrophage activation syndrome.

## Abstract (web summary)

Still’s disease is an inflammatory syndrome linked to the development of further immune dysregulation and hypercytokinaemia termed macrophage activation syndrome. Here the authors implicate the mechanistic target of rapamycin complex 1 in murine models of Still’s disease and macrophage activation syndrome, and provide associations with clinical cases in patients

## Introduction

Still’s disease (SD) is an inflammatory syndrome of unknown etiology characterized by quotidian fever, systemic inflammation, skin rash and arthritis. SD in children, also called systemic juvenile idiopathic arthritis (sJIA), was described in 1897 and adult-onset Still’s disease (AOSD) was later recognized^1,2^. The morbidity of SD is the greatest among childhood arthritides, with appreciable mortality in cases complicated by macrophage activation syndrome (MAS)^3,4^, a potentially fatal complication of overt immune dysregulation and excess cytokine production^5–7^.

Activation of the innate immune system is postulated to drive quotidian fever and systemic inflammation in the early phase of SD, engaging adaptive immune mechanisms to promote development of chronic arthritis^8^. IL-1 and IL-6 are key players in SD and antagonists of these cytokines are now approved treatments^8–10^. However, biologic agents targeting these cytokines are not uniformly effective and their availability varies in different parts of the world. It is also unclear why a subset of patients with SD (typically 10–40%) develop MAS, a process seemingly propagated by a different set of cytokines, including IL-18 and interferon (IFN)-γ^11,12^. While many questions remain regarding the pathogenesis of SD and MAS, the need for additional therapeutic options is indisputable.

In this study, we characterize the immunologic features of SD and MAS using several mouse models and complementary human studies. We show that metabolic regulator mechanistic target of rapamycin complex 1 (mTORC1) activation is essential to the development of systemic inflammation and arthritis in a murine model of SD. We find that excess mTORC1 activation in mice is sufficient to drive the development of MAS and hemophagocytosis. We demonstrate relevance of these findings in humans by analyzing transcriptomic datasets and tissue sections from patients with SD.

## Results

### IL-1 induces mTORC1 activation in a murine model of SD

**Fig. 1:**
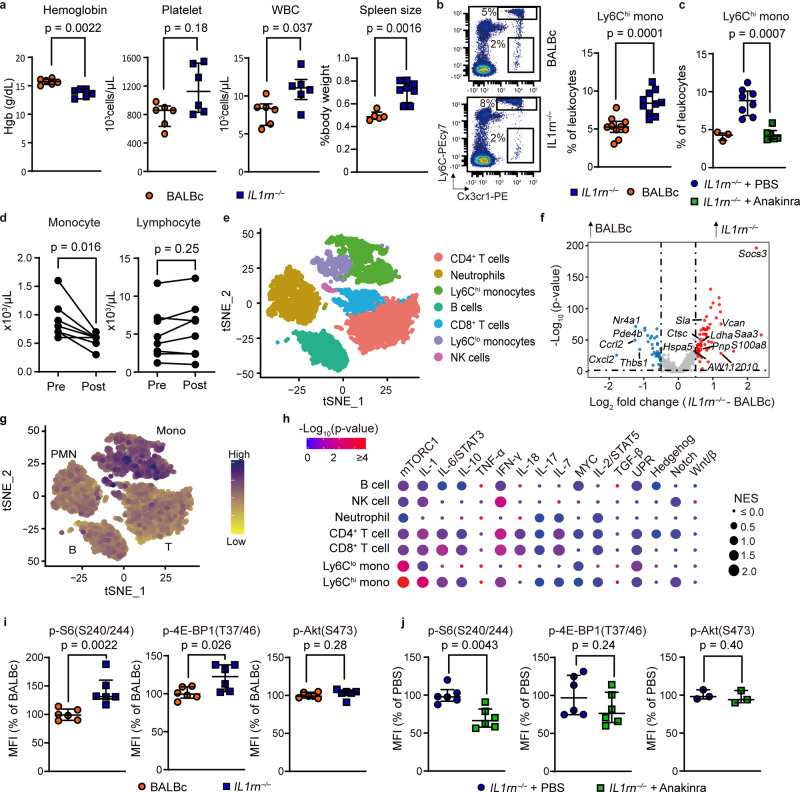
Monocytosis and enhanced mTORC1 signaling in IL-1-mediated inflammation. **a** Complete blood count in BALBc (*n* = 5) and IL1rn^−/−^ mice (*n* = 6 per group), and spleen size in BALBc (*n* = 6) and IL1rn^−/−^ mice (*n* = 8). **b** Flow cytometry quantification of circulating Ly6C^hi^ monocytes in BALBc and IL1rn^−/−^ mice at baseline (*n* = 10 per group) and (**c**) after anakinra treatment for 2 weeks (WT, *n* = 3; IL1rn^−/−^ PBS, *n* = 8; IL1rn^−/−^ + anakinra, *n* = 6). **d** Peripheral blood monocyte and lymphocyte count in sJIA patients pre-treatment and 2 weeks after initiation of anakinra (*n* = 8). **e** t-SNE display of leukocyte clustering derived from single-cell RNA-seq of peripheral blood cells from BALBc and IL1rn^−/−^ mice (4 mice pooled per group). **f** Volcano plot of differentially expressed genes in Ly6C^hi^ monocytes of WT and IL1rn^−/−^ mice. **g** Density plot of Hallmark mTORC1 gene set expression using AddmoduleScore. **h** Cluster plot of gene set enrichment in peripheral blood leukocyte subsets of BALBc and IL1rn^−/−^ mice based on single-cell RNA-seq (4 mice pooled per group). **i** Phospho-flow analysis of mTOR substrates in bone marrow Ly6C^hi^ monocytes from WT and IL1rn^−/−^ mice at baseline (*n* = 6 per group) and (**j**) after daily anakinra treatment for 2 weeks (*n* = 6 per group for phospho-S6 and phospho-4EBP1; *n* = 3 for phosphor-Akt). Data in (**a**, **b**, **c**, **i**, and **j**) were pooled from 2 to 3 independent experiments. Mice were 8–9 weeks old for all experiments. Statistical analyses (all two-sided): Mann–Whitney *U* test (**a**, **b**, **c**, **d**, **i**, **j**); Wilcoxon signed-rank test with Bonferroni correction (**f**). Median and error bars representing interquartile range are displayed in (**a**, **b**, **c**, **i**, **j**). Source data are provided as a Source Data file.

To model IL-1-driven inflammation in SD, we studied mice deficient in the endogenous IL-1 receptor antagonist (IL1rn^−/−^ mice)^13^. In addition to arthritis, by 6 weeks of age these mice developed features of systemic inflammation reminiscent of SD, including leukocytosis, thrombocytosis, anemia and hepatosplenomegaly (Fig. 1a). Further, like patients with SD^14^, IL1rn^−/−^ mice exhibited marked expansion of inflammatory (Ly6C^hi^) monocytes in the peripheral blood and bone marrow (Fig. 1b and Supplementary Fig. 1a–d). Neutrophils were increased in the peripheral blood but not bone marrow while the proportions of residential (Ly6C^lo^) monocytes and T cells were unaffected (Supplementary Fig. 1a–d). We observed an expansion of myeloid progenitors in the bone marrow of IL1rn^−/−^ mice as well as augmented differentiation of bone marrow mononuclear cells to macrophages in vitro (Supplementary Fig. 1e, f). Correspondingly, IL-1β augmented the differentiation and expansion of monocytes from myeloid progenitors in vitro (Supplementary Fig. 1g). Monocytosis was reversed by administration of recombinant human IL-1 receptor antagonist (anakinra) and recurred upon treatment withdrawal (Fig. 1c and Supplementary Fig. 2a, b). Patients with new-onset SD treated with anakinra showed a parallel reduction of circulating monocytes and improvement in laboratory features of inflammation (Fig. 1d and Supplementary Fig. 2c).

We next performed single-cell RNA sequencing analysis on circulating leukocytes from IL1rn^−/−^ mice and wild-type BALBc controls (Fig. 1e and Supplementary Fig. 3a–c). We examined differential gene expression and utilized gene set enrichment analysis (GSEA) to explore the transcriptomic landscape of immune cell subsets (Supplementary Data 1 and 2). In addition to the expected enhancement of IL-1 signaling, GSEA identified prominent activation of the mTORC1 pathway in monocytes from IL1rn^−/−^ mice (Fig. 1g, h and Supplementary Fig. 3d, e). Among the highly upregulated genes in IL1rn^−/−^ monocytes were *Socs3*, *Saa3,* and *S100a8* (Fig. 1f and Supplementary Data 1). Enhanced expression of these genes has been described in patients with SD^15,16^.

The two mTOR complexes function to integrate environmental input and regulate cellular metabolism^17^. mTORC1 plays an essential role in nutrient sensing and immune signaling^18^. To quantify mTOR activity, we performed intracellular phospho-flow staining for the mTORC1 substrates 4E-BP1 (Thr37/46) and ribosomal S6 kinase 1 (S6K1; read out by phospho-S6 Ser240/244), and the mTORC2 target Akt (Ser473). Ly6C^hi^ monocytes from IL1rn^−/−^ mice demonstrated increased S6 phosphorylation compared to controls that reversed with anakinra treatment (Fig. 1i, j and Supplementary Fig. 4a). The mTORC1-S6K1 axis was preferentially activated in monocytes from IL1rn^−/−^ mice, since augmented phosphorylation of S6 was not observed in neutrophils or lymphocyte subsets (Supplementary Fig. 4b). By contrast, the phosphorylation of Akt in all cell subsets was minimally affected by IL1rn deficiency or anakinra treatment. In vitro stimulation of myeloid progenitor cells with IL-1β also induced S6 phosphorylation and augmented cell expansion and monocyte differentiation (Supplementary Fig. 4c, d). Inhibitors of mTORC1 (rapamycin) or S6K1 (SL0101-01) abrogated these effects (Supplementary Fig. 4d).

### mTORC1 inhibition reverses systemic inflammation in IL1rn^−/−^ mice

**Fig. 2:**
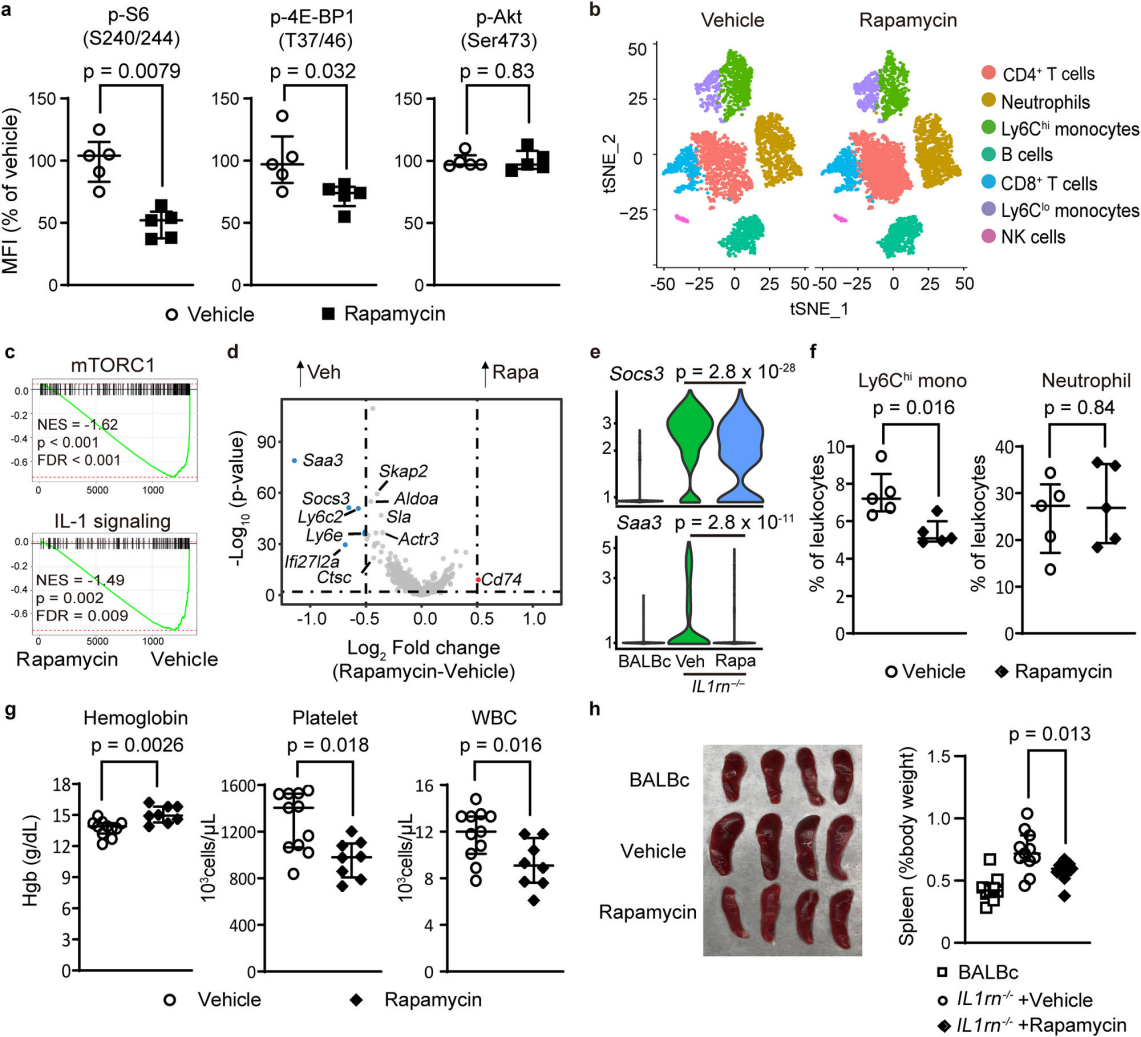
mTORC1 inhibition attenuates features of systemic inflammation in IL1rn^−/−^ mice. **a** Phospho-flow quantification of phosphorylated S6, 4EBP1 and Akt expression in bone marrow Ly6C^hi^ monocytes from IL1rn^−/−^ mice treated with rapamycin or vehicle control (*n* = 5 per group). **b** t-SNE display of leukocyte clustering derived from single-cell RNA-seq of peripheral blood leukocytes from IL1rn^−/−^ mice treated with vehicle or rapamycin for 4 weeks (4 mice pooled per group). **c** Gene set enrichment plots of mTORC1 and IL-1 gene sets, (**d**) volcano plot of differentially expressed genes and (**e**) violin plots of *Socs3* and *Saa3* expression in peripheral blood Ly6C^hi^ monocytes from single-cell RNA-seq (*n* = 4 mice pooled per group). **f** Quantification of peripheral blood monocytes and neutrophils, (**g**) complete blood count parameters in IL1rn^−/−^ mice treated with vehicle (*n* = 11) or rapamycin (*n* = 8) for 10 weeks. **h** Spleen size in BALBc mice (*n* = 9) and IL1rn^−/−^ mice treated with vehicle (*n* = 11) or rapamycin (*n* = 9) for 10 weeks. Data in (**a**, **f**, **g**, and **h**) were pooled from 2 to 3 independent experiments. Mice were 8–9 weeks old for experiments in (**a**–**e**) and 4 weeks old for experiments in (**f**–**h**). Statistical analyses (all two-sided): Mann–Whitney *U* test (**a**, **f**, **g**, and **h**), permutation test (**c**), Wilcoxon rank sum test with Bonferroni correction (**d**, **e**). Median and error bars representing interquartile range are displayed in (**a**, **f**, **g**, **h**). Source data are provided as a Source Data file.

To evaluate whether enhanced mTORC1 signaling plays a pathologic role, we treated IL1rn^−/−^ mice with rapamycin. Rapamycin decreased the phosphorylation of mTORC1 substrates as expected (Fig. 2a). Single-cell RNA-seq analysis of peripheral blood cells revealed reduced expression of genes associated with mTORC1 and IL-1 signaling in rapamycin-treated IL1rn^−/−^ mice (Fig. 2b,c and Supplementary Fig. 5a). Data for differential gene expression and GSEA are provided in Supplementary Data 1 and 2. The enhanced expression of IL-1-inducible genes *Socs3* and *Saa3* in IL1rn^−/−^ mice was also attenuated by rapamycin treatment (Fig. 2d, e). Compared to vehicle control, rapamycin strongly suppressed the expansion of inflammatory monocytes and reversed features of inflammation including anemia, leukocytosis, and thrombocytosis (Fig. 2f, g). Plasma levels of CXCL1, a chemokine induced by IL-1, were also lowered by rapamycin treatment (Supplementary Fig. 5b). While splenomegaly in IL1rn^−/−^ mice was reduced by rapamycin treatment (Fig. 2h), the proportion of naïve T cells, memory T cells and regulatory T cells in the spleen were comparable between the groups (Supplementary Fig. 5c).

**Fig. 3:**
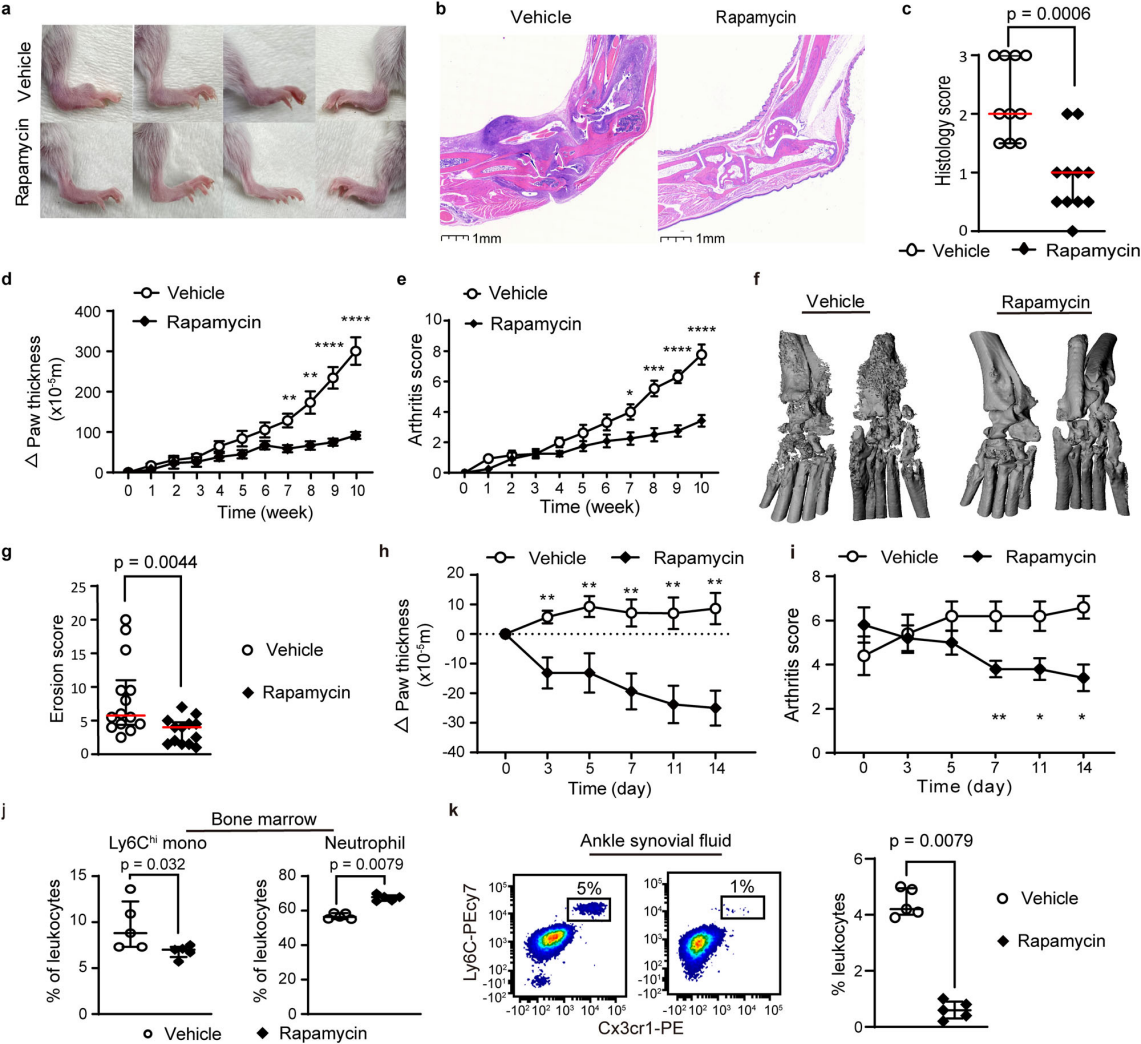
Rapamycin treatment reduces arthritis severity and bone erosion in IL1rn^−/−^ mice. **a** Representative depiction of ankle inflammation, (**b**) hematoxylin and eosin staining of ankle sections, (**c**) histologic scores of ankle arthritis (vehicle, *n* = 10; rapamycin *n* = 11), (**d**) ankle and wrist thickness (vehicle, *n* = 14; rapamycin *n* = 11), (**e**) composite arthritis score (vehicle, *n* = 14; rapamycin *n* = 11) and (**f**, **g**) micro-CT quantification of joint erosion in IL1rn^−/−^ mice treated with rapamycin or vehicle for 10 weeks. **h** Ankle and wrist joint measurements (*n* = 8 per group) and (**i**) composite arthritis score (*n* = 5 per group) in IL1rn^−/−^ mice with established arthritis treated with vehicle or rapamycin for 2 weeks. **j** Flow cytometry quantification of Ly6C^hi^ monocytes and neutrophils in the bone marrow and k) synovial fluid of IL1rn^−/−^ mice treated with vehicle control or rapamycin for 10 weeks (*n* = 5 per group). Data in (**c**–**e**, **g**–**j**, **k**) were pooled from 2 to 3 independent experiments. Mice were 4 weeks old for experiments in (**a**–**g**, **j**, **k**) and 10 weeks old for experiments in (**h**, **i**). Statistical analyses (all two-sided): Mann–Whitney *U* test (**c**, **g**, **j**, **k**); Student’s *t* test (**d**, **e**, **h**, **i**). *p < 0.05, **p < 0.01, ***p < 0.001, ****p < 0.0001. Median and error bars representing interquartile range are displayed in (**c**, **g**, **j**, **k**). Mean and error bars representing standard errors are displayed in (**d**, **e**, **h**, **i**). Source data are provided as a Source Data file.

**Fig. 4:**
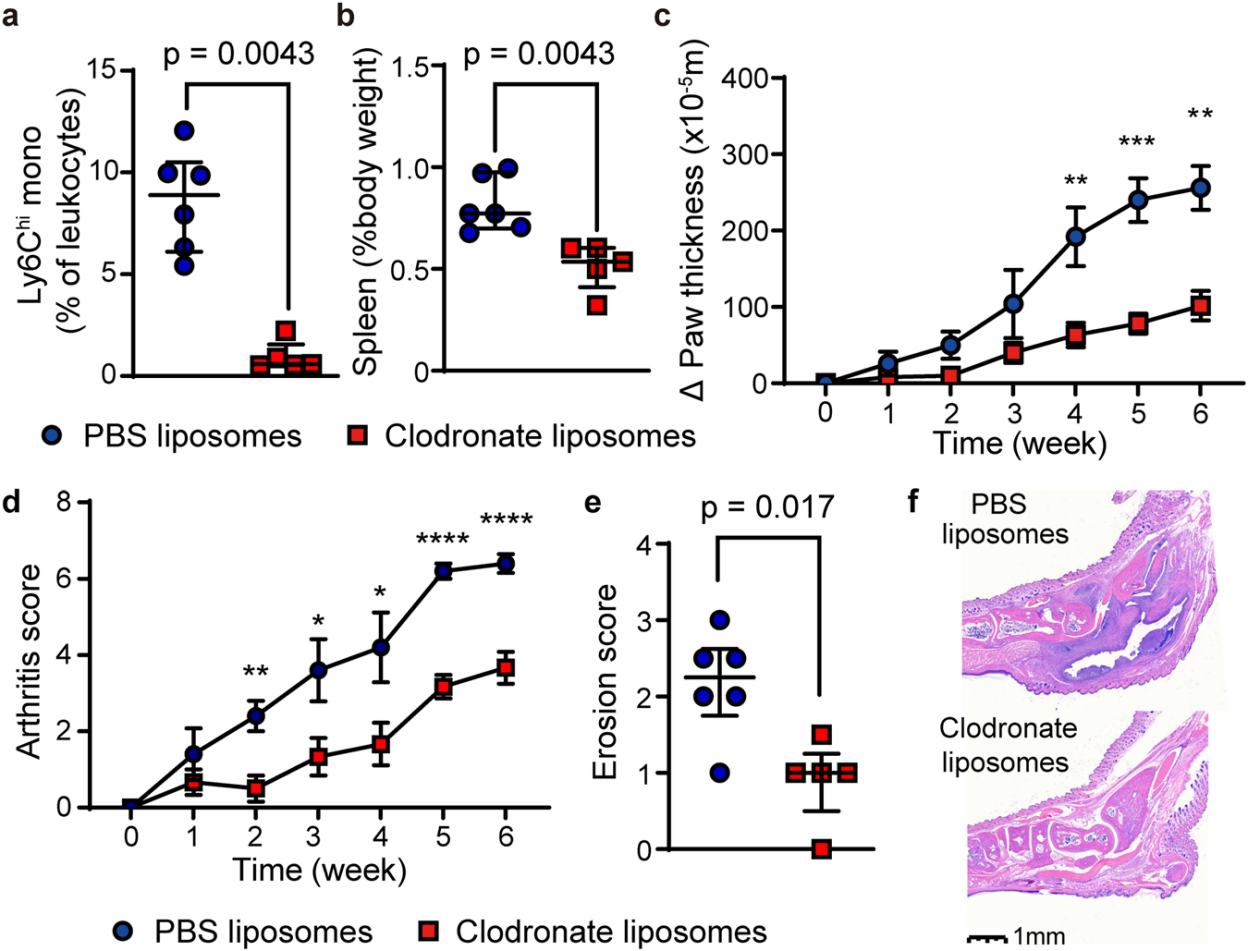
Phagocyte depletion reduces arthritis severity and bone erosion in IL1rn^−/−^ mice. **a** Peripheral blood monocyte count, (**b**) spleen size, (**c**) ankle joint thickness, (**d**) composite arthritis score, (**e**) bone erosion score (micro-CT) and (**f**) representative depiction of joint histology in IL1rn^−/−^ mice treated with PBS-liposomes (*n* = 6) or clodronate-liposomes (*n* = 5) for 6 weeks. Data in (**a**, **b**, **d**, **e**, **f**) were pooled from 2 independent experiments. Mice were 6 weeks old for experiments in (**a**–**f**). Statistical analyses (all two-sided): Mann–Whitney *U* test (**a**, **b**, **e**); Student’s *t* test (**c**, **d**). *p < 0.05, **p < 0.01. Median and error bars representing interquartile range are displayed in (**a**, **b**, **e**). Mean and error bars representing standard errors are displayed in (**c**, **d**). Source data are provided as a Source Data file.

Importantly, rapamycin suppressed the development of joint inflammation and bone erosion in IL1rn^−/−^ mice (Fig. 3a–g). The anti-inflammatory effect of mTOR inhibition was also evident in 8–10 week-old IL1rn^−/−^ mice with established arthritis (Fig. 3h, i). In addition to restricting the expansion of peripheral blood monocytes, rapamycin treatment reduced the number of Ly6C^hi^ monocytes in the bone marrow and synovial fluid of IL1rn^−/−^ mice (Fig. 3j, k). These findings and the earlier observation of prominent mTORC1 signaling in monocytes together suggest a potential pathogenic role of monocytes in this model. Indeed, depletion of monocytes using clodronate liposomes suppressed the development of splenomegaly and arthritis in IL1rn^−/−^ mice (Fig. 4a–f). The levels of IL-1β and CXCL1 were not affected by clodronate liposome treatment (Supplementary Fig. 5d). Taken together, these data illustrate the therapeutic potential of targeting monocytes and mTORC1 signaling for IL-1-mediated systemic inflammation.

### mTORC1 signature predicts treatment response in SD

**Fig. 5:**
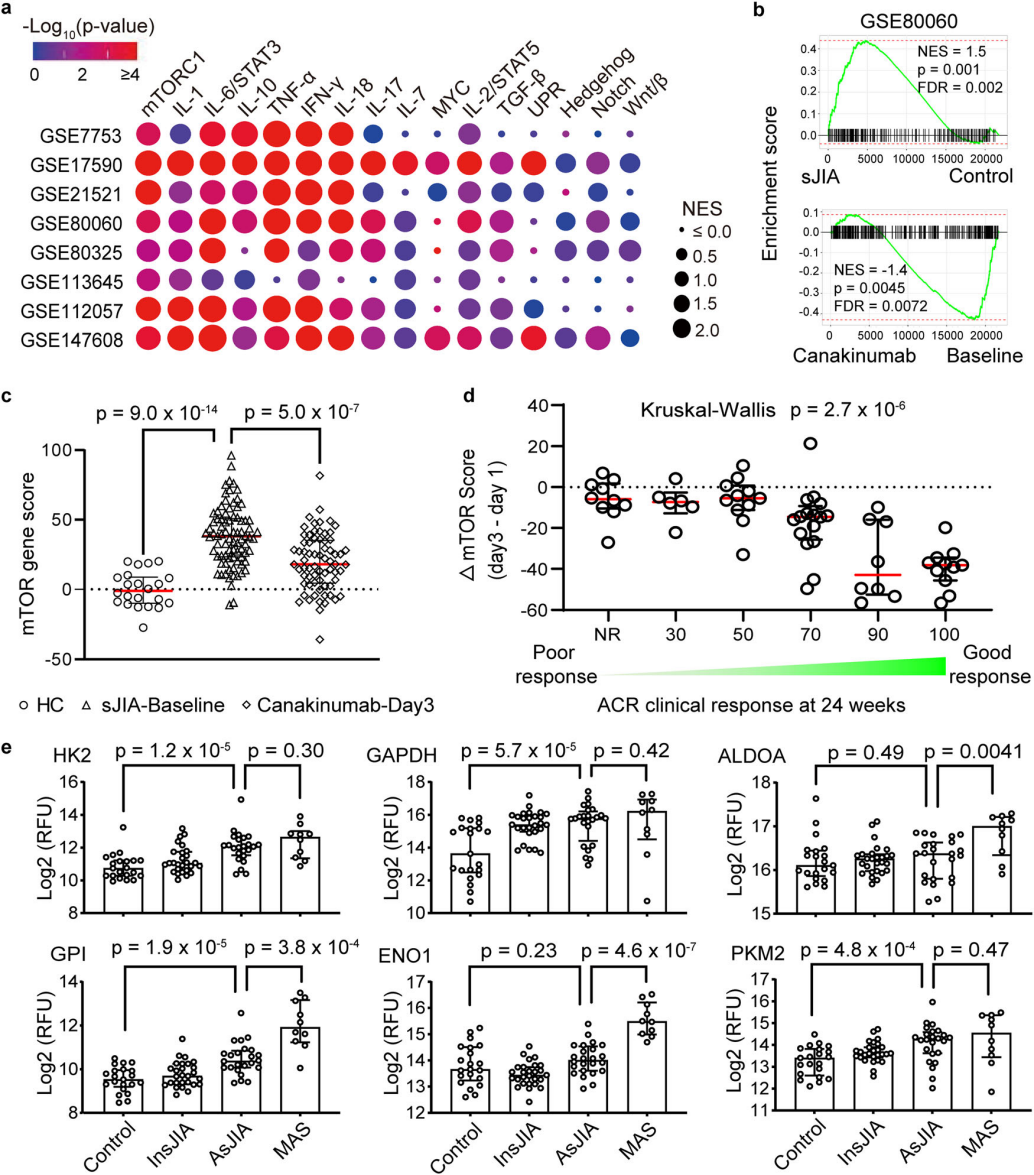
mTORC1 signature in Still’s disease correlates with disease severity and treatment response. **a** Cluster plot of gene set enrichment analysis comparing healthy controls and patients with sJIA and AOSD using in publicly available transcriptomic dataset. **b** Gene set enrichment plot of Hallmark mTORC1 gene set and (**c**) calculation of mTOR gene set score from healthy controls (*n* = 22), sJIA patients at baseline (*n* = 82) and sJIA patients 3 days after canakinumab treatment (*n* = 69). **d** Stratification of mTOR gene score before treatment and 3 days after canakinumab based on clinical response after 24 weeks. Clinical response was stratified according to American College of Rheumatology (ACR) Score: ACR 0/Non-responders (NR, *n* = 10), ACR30 (*n* = 6), ACR50 (*n* = 11), ACR70 (*n* = 16), ACR90 (*n* = 8), ACR100 (*n* = 11). **e** Levels of glycolytic enzymes from serum proteomics analysis of healthy controls (*n* = 21), sJIA patients with inactive (*n* = 27) or active disease (*n* = 24), and sJIA patients with MAS (*n* = 10). Transcriptomic data for (**b**–**d**) are derived from GEO data series GSE80060 while proteomics data in (**e**) were derived from Chen et al.^20^. Statistical analyses (all two-sided): permutation test (**a**, **b**), Mann–Whitney *U* test (**c**, **e**), Kruskal–Wallis one-way analysis of variance (**d**). Median and error bars representing interquartile range are displayed in (**c**–**e**). Source data are provided as a Source Data file.

To investigate whether excess mTORC1 signaling is associated with SD, we identified 8 transcriptomic studies that compared patients with SD and healthy controls (Supplementary Table 1). Except for one dataset on AOSD, most of these studies focused on children with sJIA. GSEA revealed consistent enrichment of the mTORC1 pathway in all datasets (Fig. 5a and Supplementary Table 2). Gene signatures that reflect IL-1, IL-6, IL-18 and IFN-γ signaling were also consistently detected in patients with SD (Fig. 5a). These inflammatory mediators have been implicated in the pathogenesis of SD and they all induced mTORC1 signaling in CD14^+^ monocytes from healthy volunteers (Supplementary Fig. 6a), suggesting that mTORC1 integrates the proinflammatory effects of these cytokines. The enrichment of mTORC1-regulated genes in children with sJIA was significantly stronger than in other childhood arthritides (Supplementary Fig. 6b, c).

We focused our analysis on the landmark trial of canakinumab (monoclonal anti-IL-1β) for sJIA from which early transcriptomic data (baseline and 3 days post-treatment) and long-term treatment response data (24 weeks) are publicly available. We calculated a standardized mTORC1 gene set score based on the expression of leading-edge genes from GSEA. In addition to distinguishing patients with sJIA from healthy controls (Fig. 5b), the mTORC1 signature correlated with treatment response to canakinumab (Fig. 5c). Patients with the best long-term clinical response after 24 weeks (based on American College of Rheumatology criteria)^10^ displayed a particularly marked reduction of the mTORC1 gene signature 3 days after treatment initiation (Fig. 5d and Supplementary Fig. 6d, e). By contrast, patients with minimal transcriptional changes in the mTORC1 gene signature after 3 days generally showed suboptimal long-term clinical response.

Similar patterns were observed with gene sets that reflect IL-1 and IL-18 signaling, in keeping with the proinflammatory role of these cytokines in SD and their ability to activate mTORC1 (Supplementary Fig. 7a–d). Illustrating the specificity of these findings, expression of an unrelated gene set (Hallmark unfolded protein response pathway, as an example) did not show enrichment in patients with SD or correlation with treatment response (Supplementary Fig. 7e, f). These data provide evidence for heightened mTORC1 activation in SD and highlight the potential of the mTORC1 gene signature as a predictor of treatment response.

In line with the role of mTORC1 as a driver of cellular metabolism, genes that encode glycolytic enzymes including ALDOA, ENO1, GAPDH, GPI and HK2 are among the targets regulated by mTORC1^19^. Upregulated expression of these genes was often observed in patients with SD across the different studies (Supplementary Fig. 7g). Supporting these findings, analysis of recently published serum proteomics data revealed that increased levels of several glycolytic enzymes was associated with active sJIA (Fig. 5e)^20^. This pattern was particularly striking in sJIA complicated by MAS, a hyperinflammatory state characterized by cytokine storm, hemophagocytosis, and multiorgan system dysfunction^21,22^.

### Excess mTORC1 activation drives MAS and hemophagocytosis

**Fig. 6:**
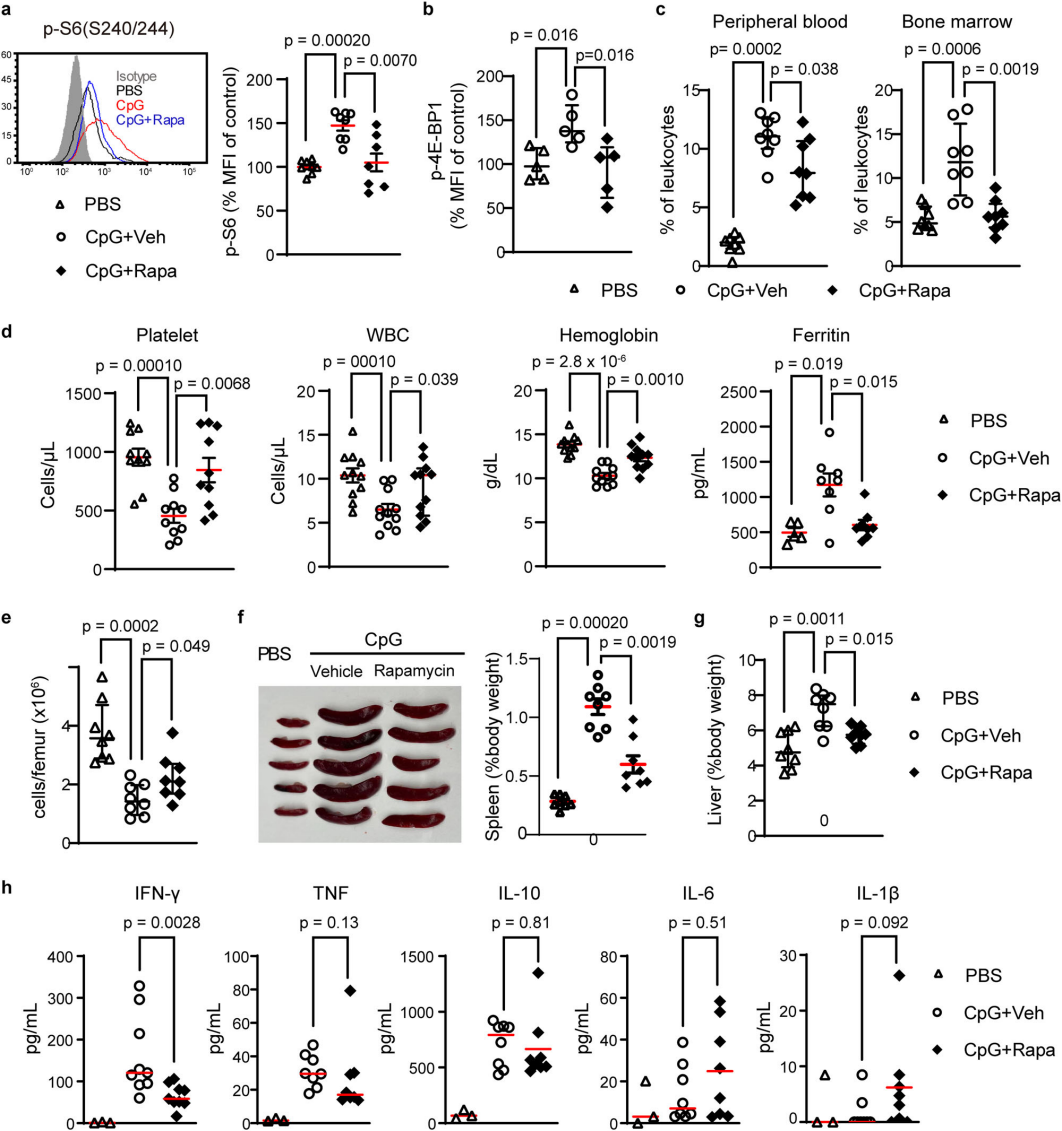
mTORC1 activation drives CpG-induced macrophage activation syndrome. **a** Phospho-S6 staining (*n* = 8 per group) and (**b**) phospho-4EBP1 staining (*n* = 5 per group) in bone marrow Ly6C^hi^ monocytes, (**c**) flow cytometry quantification of peripheral blood and bone marrow Ly6C^hi^ monocytes (*n* = 8 per group), (**d**) hematologic parameters (*n* = 11 per group) and ferritin levels (PBS, *n* = 5; CpG + Vehicle, *n* = 8; CpG + Rapamycin, *n* = 8), (**e**) absolute bone marrow cell count (*n* = 8 per group), (**f**) representative depiction and quantification of spleen size (*n* = 8 per group), (**g**) liver size (*n* = 8 per group), and (**h**) plasma cytokine levels (PBS, *n* = 3; CpG + Vehicle, *n* = 8; CpG + Rapamycin, *n* = 8), in PBS-treated or CpG DNA-treated C57BL/6 mice (50 µg every 2 days x 5 doses) given daily rapamycin or vehicle control. Data in (**a**, **b**) were normalized to the mean fluorescence intensity (MFI) of the PBS group. Mice were 8 weeks old and data from all panels were pooled from 2 to 3 independent experiments. Statistical analyses (all two-sided): Mann–Whitney *U* test (all panels). Median and error bars representing interquartile range are displayed in (**a**–**h**). Source data are provided as a Source Data file.

We hypothesized that overwhelming mTORC1 activation potentiates the development of MAS. To test this hypothesis, we examined the role of mTORC1 in a murine model of MAS induced by repeated exposure to the Toll-like receptor 9 ligand CpG DNA^23^. Development of cytopenia, hyperferritinemia and hepatosplenomegaly in CpG-treated mice coincided with an expansion of Ly6C^hi^ monocytes and enhanced mTORC1 signaling in these cells (Fig. 6a–c). Rapamycin treatment restored normal levels of mTORC1 activation, lessened the severity of cytopenia, hyperferritinemia, and hepatosplenomegaly (Fig. 6a–g), and downregulated the production of IFN-γ, an important cytokine in the pathogenesis of MAS (Fig. 6h). These findings collectively suggest a pathogenic role of mTORC1 in CpG-induced MAS.

**Fig. 7:**
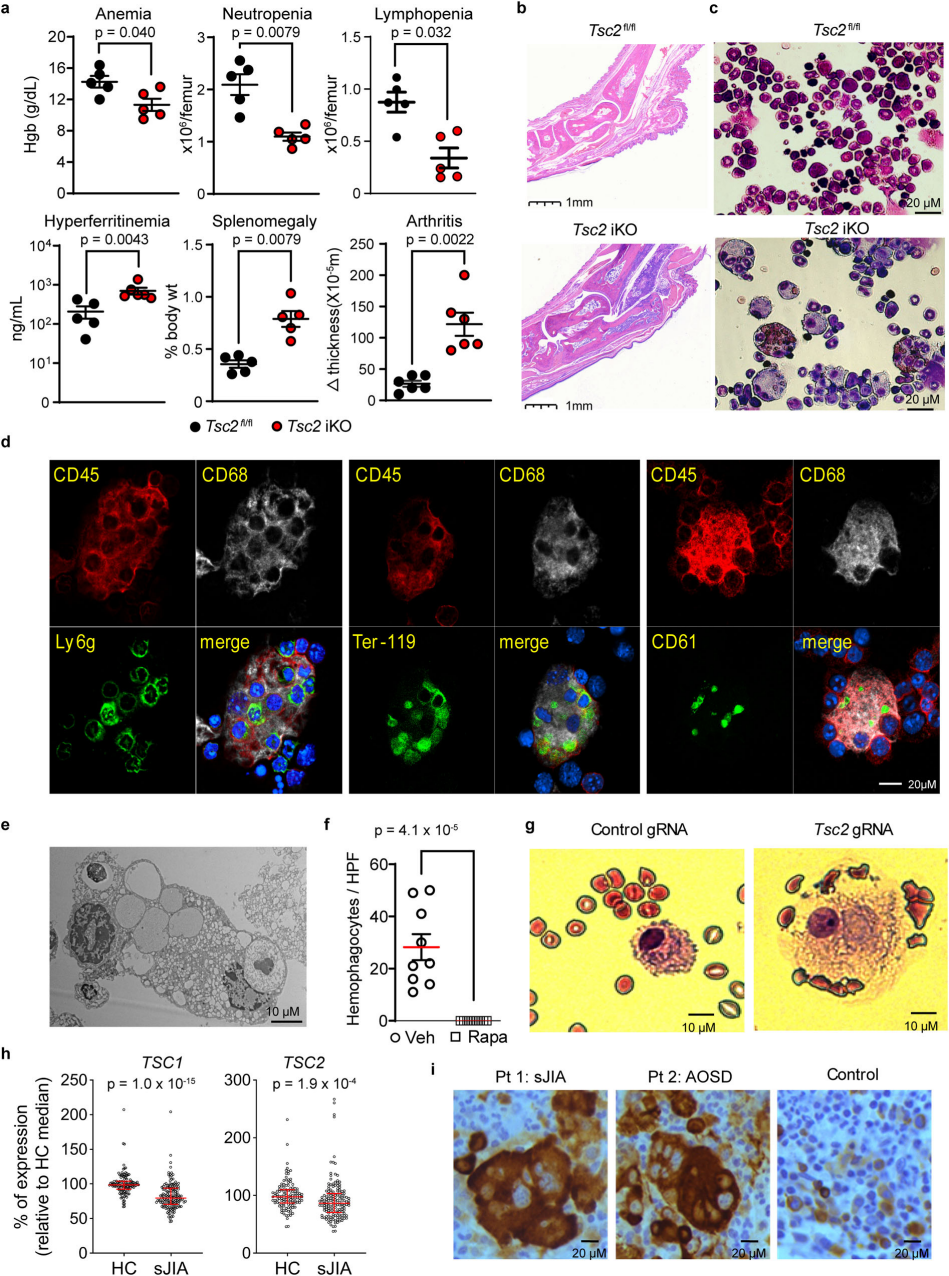
Unrestricted mTORC1 activation drives the development of hemophagocytosis and macrophage activation syndrome. **a** Hemoglobin levels (*n* = 5 per group), bone marrow cell count (*n* = 5 per group), plasma ferritin levels (Tsc2 ^fl/fl^, *n* = 5, Tsc2 ikO, *n* = 6), spleen weight (*n* = 5 per group), and change in ankle joint thickness (*n* = 6 per group) and (**b**) representative ankle pathology (H&E stain) in Tsc2 iKO mice and control mice. **c** Wright-Giemsa staining of bone marrow leukocytes from Tsc2 iKO and control mice. **d** Confocal microscopy and **e** electron microscopy of hemophagocytes from the bone marrow of Tsc2 iKO mice. **f** Quantification of bone marrow hemophagocytes in Tsc2 iKO mice treated with rapamycin or vehicle control (3 slides analyzed for each mouse; *n* = 3 per group). Mice were 6–8 weeks of age and analyses in (**a**–**f**) were performed 3 weeks after poly I:C treatment to induce Tsc2 deletion. **g** Wright-Giemsa staining of human monocytes with targeted disruption of *Tsc2* by CRISPR/Cas9 cultured with M-CSF for 14 days. **h** Pooled transcriptomic analysis of TSC1 and TSC2 expression in peripheral blood cells from patients with sJIA (*n* = 154) and healthy controls (*n* = 120). Data are compiled from GEO deposits GSE80325, GSE80060, GSE7753, GSE21521, GSE17590 and GSE112057. **i** Immunohistochemistry of phospho-S6 (S240/244) on bone marrow section from patients with Still’s disease and controls. Data in (**a**, **f**) were pooled from 2 to 3 independent experiments. Images in (**b**–**e**, **g**) are representative of 2 independent experiments. Images in (**i**) are derived from one experiment. Statistical analyses (all two-sided): Mann–Whitney *U* test (**a**, **f**, **h**). Median and error bars representing interquartile range are displayed in (**a**, **f**, **h**). Source data are provided as a Source Data file.

To evaluate whether persistent mTORC1 signaling alone is sufficient to trigger the pathology of SD and MAS, we generated inducible Tsc2 KO mice (Tuberous sclerosis complex 2 iKO; MxCre Tsc2 ^fl/fl^). The Tsc complex negatively regulates mTORC1 and disruption of the complex results in unopposed mTORC1 activation^24,25^. Mice with induced deletion of *Tsc2* spontaneously developed arthritis and an MAS-like syndrome characterized by cytopenia, elevated ferritin levels, and hepatosplenomegaly (Fig. 7a, b). Remarkably, examination of the bone marrow of Tsc2 iKO mice showed fulminant hemophagocytosis (Fig. 7c), a hallmark finding of MAS. Confocal microscopy confirmed internalization of blood elements including erythrocytes (Ter119+), neutrophils (Ly6G+) and platelets (CD61+) by CD68^+^ macrophages (Fig. 7d). Electron microscopy of these cells further delineated large vacuoles that contained intact cells and digested remnants (Fig. 7e). mTORC1 signaling was required for these observations as rapamycin treatment fully prevented bone marrow hemophagocytosis (Fig. 7f).

Bone marrow transplant using Tsc2 iKO mice and CD45.1^+^ wild-type mice demonstrated that the development of hemophagocytosis required Tsc2 deletion in hematopoietic cells (Supplementary Fig. 8a). We generated Cre/ERT2 Tsc2^fl/fl^ mice and found that tamoxifen-induced deletion of Tsc2 in sorted myeloid progenitors and Ly6C^hi^ monocytes was sufficient to induce hemophagocytosis in vitro (Supplementary Fig. 8b). In line with these findings, CRISPR/Cas9-mediated deletion of *TSC2* in human CD14^+^ monocytes induced differentiation into large hemophagocytic histiocytes (Fig. 7g and Supplementary Fig. 8c, d). While mutations in *TSC2* or its binding partner *TSC1* have not been described in SD, pooled analysis of available transcriptomic studies revealed that expression of both genes were significantly downregulated in PBMC from children with sJIA (Fig. 7h).

Finally, we searched for evidence of mTORC1 activation in hemophagocytic histiocytes from patients with MAS. Immunohistochemistry and immunofluorescence of bone marrow cells from 3 patients with SD-associated MAS showed prominent mTORC1 signaling in hemophagocytic histiocytes as suggested by phospho-S6 staining (Fig. 7i and Supplementary Fig. 8e). Similar observations were noted in patients with MAS in the setting of viral infection (Supplementary Fig. 8f), suggesting that enhanced mTOR1 signaling may be a shared feature of hemophagocytic syndromes.

## Discussion

SD is an intriguing inflammatory syndrome that bridges innate and adaptive immunity^8^. Myeloid cells and multiple cytokines have been implicated in SD but how they contribute to disease pathogenesis and why some patients develop MAS remain unresolved^26^. Using a combination of murine models and human studies, our work illustrates a role of mTORC1 signaling that connects the spectrum of inflammation in SD and MAS. These findings have direct therapeutic implications, as inhibitors of mTOR are already in clinical use.

Recent studies have revealed important roles of IL-1, IL-6, IL-18, and IFN-γ in SD and MAS^9–12,16,27,28^. While the IL1rn^−/−^ model does not directly address the role the other proinflammatory mediators, our survey of human transcriptomic data revealed consistent activation of mTORC1 in parallel with these cytokine signatures. We propose that mTORC1 may serve as a nexus that integrates signals from these cytokine pathways. Supporting this hypothesis, the pathology of IL1rn deficiency and CpG-induced MAS was largely abrogated by rapamycin treatment while unrestricted activation of mTORC1 in Tsc2 KO mice was sufficient to elicit an SD-like syndrome, including both arthritis and fulminant MAS.

As the link between immunometabolism and inflammation is increasingly recognized. mTORC1 has been implicated in complex inflammatory diseases including systemic lupus erythematosus (SLE), sarcoidosis and Castleman’s disease^29,30^. While biologics that target IL-1 and IL-6 have improved the outcome of SD, these agents are not uniformly effective, and frequent injections or infusions remain a challenge, especially for young children^31^. Further, blockade of these cytokines does not reliably prevent the development of MAS in patients with SD. Orally available mTOR inhibitors such as sirolimus and everolimus are currently used for transplant rejection, vascular anomalies, and oncologic indications^32,33^. Supporting the potential benefit of mTOR inhibition in SD, a recent case report demonstrated successful management with sirolimus of a patient with severe SD refractory to other treatments^34^.

The downstream mechanism through which mTORC1 controls the inflammatory process in SD and MAS requires further investigation. Previous studies have shown that challenging wild-type mice with TLR agonists can recapitulate key features of infection-associated MAS^35,36^. Wang and colleagues illustrated that glycolytic metabolism is responsible for the features of MAS triggered by sequential treatment with TLR3 and TLR4 agonists^36^. It is possible that the pathology of TLR-induced MAS is also mediated via mTORC1. Alternatively, mTORC1 may modulate the differentiation and function of monocytes^37^. Expansion of monocytes in the peripheral blood was associated with active disease in both patients with SD and IL1rn^−/−^ mice. Single-cell RNA-seq revealed prominent mTORC1 signaling in monocytes from IL1rn^−/−^ mice and clodronate treatment ameliorated both arthritis and systemic inflammation. Furthermore, overt activation of mTORC1 in monocytes was sufficient to drive the development of hemophagocytic histiocytes in a cell-intrinsic manner. These data suggest that monocytes may be another potential therapeutic target in SD and MAS.

A limitation of this study is the focus on myeloid cells and innate immunity. Adaptive immunity is thought to be involved in the chronic arthritis of SD, and T lymphocytes are required for the development of arthritis in IL1rn^−/−^ mice^8,38^. SD-associated MAS shares many clinical features with primary HLH caused by mutations that disrupt the lytic function of T lymphocytes and natural killer cells, leading to a vicious cycle of reciprocal immune activation and cytokine production by lymphocytes and macrophages^39^. mTORC1 inhibition may interfere with the inflammatory cascade of SD and MAS by skewing T cell differentiation^40^. Glycolytic metabolism governed by mTORC1 signaling promotes the differentiation of effector cells, while blockade of mTORC1 diverts the differentiation program towards memory T cells and regulatory T cells^41,42^. This immunomodulatory function is considered to be the likely mechanism for the beneficial effects of mTOR inhibitors in autoimmune and immunodysregulatory diseases. The use of oral mTOR inhibitors was associated with clinical improvement in clinical trials for SLE and rheumatoid arthritis^43,44^. It is noteworthy that rapamycin was not effective in the perforin-deficiency model of primary HLH^45^, which is dependent on CD8^+^ T cells. Further studies are needed to delineate the differential impact of mTOR inhibition in immune cell compartments in SD and primary HLH.

Up to one-third of patients with SD-associated MAS possess a deleterious variant in genes linked to primary HLH^46^. Although a connection between human mutations in TSC2 and MAS is not established, analysis of published transcriptomic datasets suggests decreased expression of TSC1 and TSC2 in some patients with SD. These findings implicate reduced control of mTORC1 activation as a potential susceptibility factor for MAS development in SD. Since the available transcriptomic dataset are focused on SD without MAS, in-depth profiling of MAS in the peripheral blood and affected tissues (i.e. bone marrow and liver) are needed to further evaluate the pathogenic role of mTORC1. Future studies are also needed to understand the relative contributions of genetic and environment factors to mTOR activation in SD and MAS.

Using a combination of murine models and human studies, our study illustrates a role of mTORC1 in the pathophysiology of SD. These findings connect the biology of SD and MAS directly to conserved metabolic pathways and introduce mTORC1 as potential therapeutic target.

## Methods

### Study design and approval

All experimental procedures on animals were conducted in accordance with the standards of Institutional Animal Care and Use Committee (IACUC) and approved by Brigham and Women’s Hospital and/or Boston Children’s Hospital. Approval for human subject research and waiver of consent were granted by Institutional Review Board (IRB) of Massachusetts General Hospital (Protocol: 2017P000255) and Boston Children’s Hospital (Protocol: P00005723).

### Human subjects

Data on hematologic parameters and inflammatory markers in sJIA patients were collected at Boston Children’s Hospital. For patients with sJIA, informed consent was provided by participants or legal guardians and assent was obtained from patients when appropriate. Retrospective chart review was performed to identify patients with new-onset sJIA diagnosed between January 2016 and December 2019. Deidentified laboratory data were collected from 8 subjects with new-onset sJIA (4 males, 4 females; median age = 5 years) without evidence of MAS at the time of presentation. To avoid selection bias of using laboratory data from a single time point, pre-treatment data represent an average from 3 time points before treatment with the recombinant IL-1 receptor antagonist anakinra while post-treatment data represent the average of 2–3 time points within the first month of anakinra treatment.

For evaluation of bone marrow pathology in patients with MAS, patients were identified through an internal database at Massachusetts General Hospital. Demographic and clinical data from these subjects are provided in Supplementary Table 3. Control bone marrow sections were obtained from two individuals who underwent bone marrow biopsy for anemia or hypogammaglobulinemia. No pathologic features were identified in the biopsy of these two individuals.

### Reagents

Recombinant murine IL-1β, M-CSF (macrophage colony stimulating factor), and human IL-18 were purchased from Biolegend (San Diego, CA, USA). Human IL-1β, IL-6, IFN-γ, and murine SCF (stem cell factor) were purchased from Peprotech (Rock Hill, NJ, USA). Rapamycin was purchased from LC Laboratories (Woburn, MA, USA). Rapamycin was solubilized with Tween 80 and PEG-400 (Sigma Aldrich, St. Louis, MO, USA) as described^47^. SL0101-01 and 4OH-tamoxifen were purchased from Cayman Chemical (Ann Arbor, MI, USA). Poly I:C and Osteosoft solution were obtained from Sigma-Aldrich. Clodronate liposome and PBS liposome were purchased from ENcapsula NanoSciences (Brentwood, TN, USA). CpG was purchased from Integrated DNA Technologies (Coralville, IA, USA). Fixation and permeabilization buffer for intracellular staining were purchased from Biolegend. Tissue culture reagents and Hoechst 33342 were purchased from ThermoFisher (Waltham, MA, USA). Anakinra was manufactured by Sobi (Stockholm, Sweden). All antibodies used in this study, their sources and applied dilution are listed in Supplementary Table 4.

### Animal studies

Wild-type BALB/c, wild-type C57BL/6, CD45.1 (B6.SJL-Ptprca Pepcb/BoyJ), ubiquitin ERT2-Cre (B6.Cg-Tg(UBC-cre/ERT2)1Ejb/1J), and Mx1-Cre (B6.Cg-Tg(Mx1-cre)1Cgn/J) were purchased from Jackson Laboratories. Tsc2 ^fl/fl^ (Tsc2^tm1.1Mjg^/J) mice were kindly provided by Dr. Michael Gambello^48^ and bred with Mx1-Cre mice, ubiquitin-ERT2-Cre to generate Cre^+^Tsc2 fl/fl mice. BALB/c Il1r1^tm1Imx^/J mice (IL1rn^−/−^) were described previously^13^. Male and female mice were randomized to experimental groups with matched littermate controls. Mice were housed and bred under specific pathogen-free conditions in a vivarium at the Brigham and Women’s Hospital or Boston Children’s Hospital.

#### Poly I:C treatment

To induce deletion of Tsc2, 6 to 8-week-old Mx-Cre^+^TSC2 ^fl/fl^ mice or Tsc2 ^fl/fl^ controls were given poly I:C (250 μg i.p.) every other day for 3 doses.

#### Anakinra treatment

To study the effects of recombinant IL-1Ra treatment, IL1rn^−/−^ mice (6 weeks old) were injected intraperitoneally with 10 mg/kg anakinra or vehicle control (PBS) daily for 1 or 2 weeks.

#### Rapamycin treatment

Rapamycin was dissolved in 100% ethanol and further diluted in a vehicle solution containing 5% PEG 400 and 5% Tween 80 prior to intraperitoneal injection^47^. IL1rn^−/−^ mice (4 to 10 weeks old) were treated with rapamycin (2 mg/kg) or vehicle control daily for 2–10 weeks.

#### Clodronate-liposome treatment

IL1rn^−/−^ mice (6 weeks old, male and female) were given intraperitoneal injection of clodronate-loaded liposomes (100 μL containing 18.4 mM clodronate; twice weekly) or PBS liposomes for 6 weeks.

#### CpG DNA treatment

Wild-type C57BL/6 mice (8 weeks old, male and female) were treated with intraperitoneal injections of CpG (50 μg) or PBS every other day for 10 days.

### Bone marrow transplant

Recipient CD45.1 mice and CD45.2 Mx-Cre Tsc2^fl/fl^ mice (8 weeks old, male) were irradiated using two split doses of 500 rads and given 2 × 10^6^ bone marrow mononuclear cells from CD45.2 Mx-Cre Tsc2^fl/fl^ donors and CD45.1 donors via intravenous injection, respectively. Engraftment was confirmed by FACS analysis of PBMC after 4 weeks followed by induction of Tsc2 deletion by poly I:C.

### Cell count and cytokine quantitation

Complete blood cell count (CBC) was performed in a hematology analyzer (Sysmex, XP-300, Kobe, Japan). Plasma cytokines / chemokines were quantified using the Legendplex assay (Biolegend) according to manufacturer’s instructions. Plasma ferritin was quantified using a mouse ferritin ELISA Kit (Crystal Chem; Elk Grove Village, IL, USA).

### Flow cytometry

For surface staining, cells were stained with an optimized amount of primary antibody or the appropriate isotype control for 15 min at room temperature before washing and resuspending in PBS supplemented with 0.1% BSA. Intracellular staining was performed after surface staining using fixation and permeabilization buffer (Biolegend). Antibodies were added to cells after the permeabilization step and samples were incubated overnight on ice before analysis. For phospho-flow analysis of mTOR substrates in bone marrow monocytes, cells were directly fixed by flushing femurs with 4% paraformaldehyde to prevent changes in mTORC1 activation during the process of cell isolation. Samples were acquired using a Becton-Dickinson FACS Canto II flow cytometer and analyzed with FCS Express 5 software (De Novo Software, Pasadena, CA, USA). In all experiments, intact cells were identified by size and singlets were gated for analysis. Isotype control antibodies were used to establish gates to determine cell population and quantify mean fluorescence intensity. A centrifugation speed of 400 *g* was used during the processing of samples for flow cytometry.

### Development of hemophagocytes in vitro

Bone marrow cells were isolated from the femur of ubiquitin-ERT2-Cre Tsc2 ^fl/fl^ mice and stained with antibodies for flow sorting. After lysis of erythrocytes in ACK buffer, cells were washed in PBS supplemented with 0.1% BSA and resuspended in complete RPMI. Common myeloid progenitors (CMP, Lin-ckit+CD32^lo^CD34^hi^), granulocyte*-*monocyte *progenitors (*GMP, *Lin-ckit* + *CD32*^*hi*^*CD115*^*lo*^*), c*ommon *monocyte progenitors (*CMoP, Lin-ckit+CD32^hi^ CD115^hi^ Ly6C^hi^), and Ly6C^hi^ monocytes (ckit-CD115^hi^Ly6C^hi^) were sorted by a FACSAria^TM^ Fusion Cell Sorter (BD). Sorted cells were cultured in complete RPMI medium with stem cell factor (40 ng/mL) and 4OH-tamoxifen (1 μM) for 6 days prior to the addition of M-CSF (20 ng/mL). On day 13, whole blood (10 μL) from wild-type mice was added to the cultured cells to induce hemophagocytosis. After 24 h, hemophagocytes were imaged following Giemsa Wright staining (Sigma-Aldrich).

### ER-Hoxb8 cells

Generation of ER-Hoxb8 myeloid progenitor cells and in vitro differentiation of monocytes were described previously^37^. Wild-type ER-Hoxb8 cells were differentiated in RPMI 1640 medium supplemented with 10% fetal bovine serum (Gemini; Sacramento, CA, USA), 1% penicillin-streptomycin, 10 ng/mL recombinant mouse stem cell factor with or without recombinant mouse IL-1β (up to 100 ng/mL). In some experiments, cells were treated with rapamycin (500 nM), SL0101-01 (10 nM) or DMSO at the time of differentiation. Cells were analyzed after 4 days by flow cytometry. A centrifugation speed of 400 *g* was used for experiments using ER-Hoxb8 cells.

### Confocal microscopy

Cells were fixed and permeabilized prior to incubation with optimized amounts of primary antibodies overnight in PBS containing 2% rat or rabbit serum. Cells were then incubated with fluorophore-conjugated secondary antibodies and Hoechst nuclear dye for 2 h. After washing in PBS, cells were cytospun onto glass slides. Cover slip was applied with FluoroMount solution (Thermo Fisher). Confocal images were captured using a Zeiss LSM 800 Laser Scanning Confocal Microscope system (Oberkochen, Germany).

### Electron microscopy

Electron microscopy was performed as described^49^ with assistance from the Electron Microscopy Facility at Harvard Medical School. Bone marrow cells from Tsc2 iKO mice and control mice were fixed using 2.5% glutaraldehyde and 2.5% paraformaldehyde in 0.1 M sodium cacodylate buffer (pH 7.4) for 1 h at room temperature. After washes in 0.1 M sodium cacodylate buffer (pH 7.4), cells were incubated with 1% osmium tetroxide (OsO4)/1.5% potassium ferrocyanide (KFeCN6) for 30 min prior to sectioning. Sections (60-80 nM) were imaged with a JEOL1200EX electron microscope and images were recorded with an AMT 2k CCD camera (Peabody, MA, USA).

### Immunohistochemistry

Immunohistochemistry was performed by Specialized Histopathology Core, Dana Farber/Harvard Cancer Center. Human bone marrow sections were deparaffinized in xylene, rehydrated ethanol, washed in water before antigen retrieval with 10 mM sodium citrate. After washing with water, slides were blocked with TBS-T with 5% normal goat serum prior to overnight incubation with primary antibodies (rabbit anti-phospho S6 S240/244 or isotype control; 1:2000 dilution). Secondary antibodies (Signal Stain Boost IHC detection reagent; Cell Signaling; Ipswich, MA, USA; 1:5000 dilution) were applied for 20 min after washing. The slides were washed in 3 times with TBS and developed with horseradish peroxidase compatible DAB for 2 min (DAKO; Glostrup, Denmark). The reaction was then terminated with water and slides were counterstained with hematoxylin.

### Arthritis assessment

The degree of arthritis was graded for the paws according to a visual score system from 0 (no evidence of inflammation) to 3 (pronounced edema and erythema in the entire paw)^50,51^, while the paw thickness was measured by a dial thickness gauge (Kafer Model JZ 15, Germany). The score and change in thickness from 4 paws were combined for each time point for longitudinal analysis.

Isolated ankles were fixed in 4% paraformaldehyde for 24 h and decalcified in Osteosoft solution for 7 days. Tissue sectioning and staining were performed by iHisto Inc. The ankles were embedded in paraffin blocks, sectioned, and stained with hematoxylin and eosin. SMASH recommendations^52^ were used for histologic assessment of synovial inflammation. A score ranging from 0 (no inflammation) to 3 (severe synovitis) was assigned to each sample by two blinded investigators.

### Micro-CT scan and assessment of bone erosion

Ankles were fixed in 10% neutral-buffered formalin, followed by storage in 70% ethanol. CT scan was performed on a Scanco μCT 35 (Scanco Medical AG; Brüttisellen, Switzerland) in 70% ethanol using a voxel size of 20 μM, X-ray tube potential of 55 kVp, intensity of 0.145 Ma, and integration time of 400 ms. DICOM files were exported and 3D images were reconstructed by 3D viewer in Fiji^53^. The anterior view and posterior view of ankles were used to evaluate ankle bone erosion. A score of 0–3 was assigned to the bones in the ankle (talus bone, malleolus bone, calcaneus bone, cuneiform bone, navicular bone, cuboid bone): 0 = normal (intact bone surface), 1 = mild (minimal / early-stage bone surface defects), 2 = moderate (obvious bone surface defects), 3 = severe (loss of bone surface structure). The scoring was performed blindly by two independent investigators. Findings of slight erosions in wild-type mice might be due to natural bone surface defect or image processing. The composite erosion score was calculated from the sum of scores assigned to the ankle bones for each animal.

### GEO data analysis

RNA sequence or microarray data on sJIA and AOSD were downloaded from Gene Expression Omnibus (GEO) datasets (www.ncbi.nlm.nih.gov/geo/). The analyzed datasets are described in Supplementary Table 1. Differentially expressed genes (DEGs) dentification, heatmap of the DEGs, volcano plot and gene set enrichment analysis were performed with limma package (v3.46.0)^54^, fGSEA package (v1.16.0)^55^, complexheatmap package (v2.6.2)^56^ and ggplot2 package (v3.3.3)^57^ in R (v4.0.4)^58^. Gene sets were chosen to study major immune-related pathways and sources of all gene sets analyzed in this study are listed in Supplementary Table 5. mTORC1 gene signature score for GSE80060 was calculated from the leading-edge genes in the Hallmark mTORC1 gene set that were enriched in sJIA patients compared to healthy controls (Supplementary Table 2)^59^. A standardized score of each gene was derived based on the number of standard deviations away from the mean of control group. The mTORC1 gene set score represents the sum of standardized score for all leading-edge genes. This method was previously described for the calculation of interferon gene signature^60,61^. For analysis of TSC1 and TSC2 expression, datasets derived from PBMC and whole blood were included and expression of these genes in each patient was normalized as a percentage of the control group average from the same study.

### Single-cell RNA sequencing

Single-cell sequencing was performed by the Brigham and Women’s Hospital Single Cell Genomics Core. Whole blood was collected from BALB/c mice and IL1rn^−/−^ mice treated with rapamycin or vehicle for 10 weeks. Leukocytes were isolated from whole blood after lysis of red blood cells. Leukocytes were incubated with Mouse TruStain FcX (BioLegend) for 10 mins at 4 °C followed by incubation with lineage antibodies (B220, CD11b, Ly6G) and hashing antibodies (TotalseqB Hashtag-9, 10 and 11; Biolegend) for 30 mins at 4 °C. The cells were washed and resuspended in Cell Staining Buffer (BioLegend) for sorting by FACSAria^TM^ Fusion Cell Sorter (BD). Gates were preset to acquire 20% neutrophils (Ly6G+), 20% monocytes (CD11b+ly6G−), 20% B lymphocytes (B220 + ) and 40% cells negative for these markers (mostly T cells). This strategy was employed to capture a sufficient number of cells for each subset to allow downstream analysis. Therefore, the cell distribution from single-cell RNAseq is not expected to represent the natural distribution of peripheral blood cells in mice. Quantification of leukocyte subsets was performed separately by flow cytometry.

Sorted cells were loaded onto a Chromium chip G (10X Genomics) followed by encapsulation in a lipid droplet (Single Cell Next GEM kit V3.1, 10X Genomics). The scRNA-seq libraries were generated according to the manufacturer’s protocol. Using Illumina Novaseq, single-cell mRNA library was sequenced to an average of 30,000 reads per cell, and HTO (Cell Hashing antibodies) library sequenced to an average of 5,000 reads per cell. ScRNA-seq reads were processed with Cell Ranger v3.1^62^, including demultiplexing, alignment (using GRCm38/mm10), and barcode processing. The gene matrices were further analyzed by seurat package (v4.0.1)^63^, limma package (v3.46.0), fGSEA package (v1.16.0), complexheatmap package (v2.6.2) and ggplot2 package (v3.3.3) in R (v4.0.4).

### TSC2 deletion in human monocytes

PBMC were isolated via Ficoll-Paque Plus gradient centrifugation and residual RBCs were lysed in ACK lysing buffer. Cells were resuspended in isolation buffer (PBS with 2% FBS, 1 mM EDTA) at room temperature at a concentration of 5 × 10^7^/mL. Human monocytes were negatively isolated from PBMCs using EasySep™ Human Monocyte Isolation Kit (Stemcell technologies; Cambridge, MA, USA) following manufacturer’s instructions. Isolated monocytes were rested in RPMI 1640 (Sigma) supplemented with 10% FBS, 10 mM HEPES, 1 mM sodium pyruvate, 2500 mg/L glucose, 1% penicillin-streptomycin and 0.05 mM 2-mercaptoethanol for 1 h at room temperature.

Guide RNA targeting TSC2 (sequence:5’-GAUGGACAGGACGAUCUCAU-3’) and control scrambled sgRNA (5’-GCACUACCAGAGCUAACUCA-3’) were purchased from Synthego (Redwood City, CA, USA) and resuspended in TE buffer at a concentration of 40 μM. The selection of target sequences to maximize on-target and minimize off-target binding was aided by the web applications VBC SCORE. One microliter of sgRNA was mixed with 1 μl of 20 μM recombinant Streptococcus pyogenes Cas9 (Synthego) and incubated at 37 °C for 15 min to form ribonuclear protein complexes (RNP). Monocytes (1 × 10^6^/well) resuspended in P3 buffer (Lonza; Morristown, NJ, USA) were added to 16-well Nucleocuvette Strips (Lonza). To each reaction, 2 µl of RNPs (with TSC2 or control gRNA) were added to the cells. Nucleocuvette Strips were loaded into an Amaxa 4D Nucleofector (Lonza) and electroporated using program EA-100. Electroporated human monocyte was cultured in complete RPMI (5 × 10^5^/mL) in the presence of 50 ng/ml of human MCSF. DNA was collected from a portion of cells to analyze the efficiency of gene editing after 3 days. To assess hemophagocytosis, cells were cultured for 2 weeks (200 µL volume in 96-well plate) and human whole blood (1 µl of 1:10 dilution in PBS) was added for 24 h.

### Western blotting

Cells were collected and lysed in RIPA buffer (Thermo Fisher) supplemented with protease inhibitor and phosphatase inhibitor (Thermo Fisher) for 30 min. Cell lysate was collected after centrifugation at 12,000 rpm for 5 min at 4 °C. Protein concentration was determined by Qubit 4 Fluorometer (Thermo Fisher). Samples were separated by SDS-PAGE resolving gels and transferred to nitrocellulose membrane. After blocking with 5% milk powder, the membrane was incubated overnight with anti-TSC2 IgG (1:1000) or anti-β-Actin (1:2000) as loading control. After washing in TBS-T, cells were incubated with HRP-conjugated secondary antibodies (1:2500; anti-rabbit IgG for TSC2 and anti-mouse for Actin) for 1 h and washed further prior to development. Chemiluminescence was detected using a ChemiDoc (Bio-Rad, Hercules, CA, USA) following development with a 1:1 mixture of luminol/enhancer and peroxide solution (Bio-Rad).

### Statistics and reproducibility

For measurement of joint thickness and arthritis scores, serial measurements represent mean ± standard error of mean (SEM). For comparison of laboratory parameters in patients before and after anakinra treatment, Wilcoxon signed-rank test was used for comparison of pre- vs. post-treatment effects. For other quantitative variables, differences between two groups were analyzed by Mann–Whitney U test and Kruskal–Wallis test was used for comparison of multiple groups. Median and interquartile range are displayed unless stated otherwise. Student’s t-test was used for quantification of joint measurements and arthritis score. All tests were two-sided, and p < 0.05 was considered significant. For GSEA, *p*-values for each gene set were calculated by repeated permutation (set at 1000 for all analyses in this study)^64^. Wilcoxon ranked sum test with Bonferroni correction was used for the comparison of gene expression in scRNAseq.

Each experimental group had at least 3 biological replicates. Mice were randomly assigned to treatment groups, and efforts were made to achieve equal representation of males and females in each experiment. No statistical method was used to predetermine sample size. No data were excluded from the analyses. Scoring of joint pathology was performed blindly by two investigators. The investigators were not blinded to the outcome assessment for other experiments. Except for the clinical data and immunohistochemistry studies of bone marrow sections from patients, all results were confirmed by two or more independent experiments. Statistical analyses were performed using Prism 9.0 software.

### Reporting summary

Further information on research design is available in the Nature Portfolio Reporting Summary linked to this article.

## Supplementary information

Supplementary Information

Description of Additional Supplementary Files

Supplemental Data 1

Supplemental Data 2

Reporting Summary

## Data Availability

The Single cell RNA sequencing data used in this study are available in the NCBI Gene Expression Omnibus database under accession code GSE181243. Datasets analyzed in this study include GSE80325, GSE80060, GSE7753, GSE21521, GSE17590, GSE147608, GSE112057, and GSE113645. Source data are provided with this paper.

## Source data

Source Data
