# Supplementary material for: mTORC1 links pathology in experimental models of Still’s disease and macrophage activation syndrome
Source: Nat Commun. 2022 Nov 28;13:6915. doi: 10.1038/s41467-022-34480-6 (PMC9705324; doi:10.1038/s41467-022-34480-6)
Supplement: Supplementary file 1 — Supplementary Information [file 41467_2022_34480_MOESM1_ESM.pdf]

Supplementary Table 1. Transcriptomic studies of sJIA and AOSD in GEO repository. [1-8]

GEO ID	Pubmed ID	Groups compared (size)	Source	Technique
GSE80325	28935693	HC (n = 6), sJIA (n = 12)	whole blood	microarray
GSE80060	28115015	HC (n = 22), baseline sJIA (n = 77), post-canakinumab (n = 67)	whole blood	microarray
GSE7753	17968951	HC (n = 30), sJIA (n = 17)	PBMC	microarray
GSE21521	20576155	HC (n = 59), sJIA (n = 29), other JIA (n = 176)	PBMC	microarray
GSE17590	21173013	HC (n = 21), sJIA (n = 24)	PBMC	microarray
GSE147608	33277241	HC (n = 11), sJIA (n = 26)	monocytes	RNAseq
GSE112057	29950172	HC (n = 12), oligoarticular JIA (n = 43), polyarticular JIA (n = 46), sJIA (n = 26)	whole blood	RNAseq
GSE113645	30404814	HC (n = 3), inactive AOSD (n = 14), active AOSD (n = 10)	monocytes	microarray

Supplementary Table 2. Enrichment of Hallmark mTORC1 gene set in GEO datasets related to Still's disease.

**Gene set:**	**Hallmark mTORC1 Signaling**	**Leading edge genes**
**GEO study ID**	**GSE80060**	PLOD2, BCAT1, SLC2A1, SLC7A5, DAPP1, NFIL3, G6PD, NAMPT, MTHFD2L, IDI1, PPP1R15A, SLC2A3, SERP1, CFP, GSK3B, CD9, ACSL3, CDKN1A, TXNRD1, HMGCR, GBE1, SLA, ME1, GSR, SLC1A5, UBE2D3, ACTR2, RIT1, SLC6A6, SKAP2, HK2, GLRX, M6PR, RAB1A, P4HA1, IFRD1, MTHFD2, IDH1, PGM1, ACTR3, PDK1, TBK1, PDAP1, GCLC, CTSC, BHLHE40, AK4, STIP1, NFKBIB, ITGB2, ALDOA, PGK1, ELOVL5, SSR1, CXCR4, EGLN3, TFRC, ETF1, CORO1A, PSMD12, PSMC6, VLDLR, DDIT4, GAPDH, SLC7A11, FGL2, TRIB3, PSMA3, TUBA4A, LDHA, YKT6, GLA, NFYC, ADD3, HMBS, COPS5
group 1	Healthy controls	
group 2	sJIA pretreatment	
Enrichment Score	0.44	
Net Enrichment Score	1.50	
nominal p-value	1.18E-03	
adjusted p-value	2.35E-03	
**GEO study ID**	**GSE80325**	HK2, BCAT1, VLDLR, SLC6A6, RIT1, ALDOA, DDIT4, NFIL3, SLC2A3, IFRD1, NAMPT, ERO1A, TXNRD1, DDIT3, GAPDH, SLA, GBE1, PGM1, HMGCR, GSK3B, IFI30, CXCR4, G6PD, ENO1, P4HA1, TUBA4A, PLOD2, TPI1, GSR, NFYC, RPN1, CORO1A, CD9, BHLHE40, SLC9A3R1, ATP6V1D, CTSC, PPP1R15A, FDXR, ACTR2, SQSTM1, ITGB2, RRM2, MAP2K3, SCD, TBK1, ACSL3, CFP, ELOVL5, TMEM97, LDHA, SKAP2, PGK1, GLRX, SEC11A, UBE2D3, RAB1A, ADIPOR2, LTA4H
group 1	Healthy controls	
group 2	sJIA pretreatment	
Enrichment Score	0.32	
Net Enrichment Score	1.43	
nominal p-value	1.07E-02	
adjusted p-value	2.44E-02	
**GEO study ID**	**GSE7753**	PLOD2, RRM2, HMBS, SLC7A5, SLC2A1, NFIL3, PPP1R15A, NAMPT, CDKN1A, PSAT1, DHCR24, BUB1, CDC25A, MCM4, SCD, LDLR, SLC2A3, G6PD, TRIB3, ENO1, MCM2, PHGDH, MTHFD2L, ALDOA, BCAT1, SLC1A5, DDIT3, CFP, AURKA, MTHFD2, TFRC, PFKL, PNP, DHCR7, GSR, CCNF, DHFR, SQLE, NMT1, TM7SF2, RRP9, GPI, TPI1, NFKBIB, ATP6V1D, TUBA4A, CD9, PDK1
group 1	Healthy controls	
group 2	active sJIA	
Enrichment Score	0.39	
Net Enrichment Score	1.53	
nominal p-value	1.34E-03	
adjusted p-value	3.59E-03	
**GEO study ID**	**GSE21521**	PLOD2, HMBS, RRM2, SLC2A1, G6PD, SLC7A5, BUB1, PPP1R15A, CDC25A, NFIL3, PSAT1, SLC1A5, CDKN1A, DHCR24, LDLR, NAMPT, MCM2, TRIB3, PHGDH, ALDOA, ENO1, DHCR7, MCM4, CD9, SCD, DDIT3, SLC2A3, RRP9, MTHFD2L, CFP, GPI, TUBG1, TPI1, BCAT1, GSR, PFKL, NMT1, TFRC, TUBA4A, PNP, BHLHE40, TM7SF2, SQSTM1, IDI1, NFKBIB, DDX39A, DHFR
group 1	Healthy controls	
group 2	active sJIA	
Enrichment Score	0.47	
Net Enrichment Score	1.86	
nominal p-value	1.14E-05	
adjusted p-value	4.55E-05	

**GEO study ID**	**GSE147608**	UFM1, ITGB2, PSME3, CFP, IFI30, ADD3, LDHA, FADS2, PSMA4, SQSTM1, PSMA3, ATP6V1D, BCAT1, PNP, SLC2A3, PPA1, CYB5B, HSPE1, SORD, BTG2, GMPS, NFYC, GPI, PSMD12, ACLY, HMGCR, ALDOA, GGA2, SLC37A4, HSP90B1, P4HA1, PSMG1, NUP205, SLC9A3R1, HSPD1, ENO1, VLDLR, TRIB3, CACYBP, SC5D, NMT1, CDKN1A
group 1	Healthy controls	
group 2	sJIA pretreatment	
Enrichment Score	0.41	
Net Enrichment Score	1.63	
nominal p-value	3.19E-04	
adjusted p-value	8.51E-04	
**GEO study ID**	**GSE113645**	LDLR, SLC2A3, BCAT1, PNP, ME1, SLA, NFIL3, PSMD12, IDH1, RIT1, DHCR24, CYP51A1, DHFR, LDHA, IDI1, STARD4, RRM2, PSMA3, EDEM1, SRD5A1, XBP1, ATP6V1D, UBE2D3, ACACA, RAB1A, ETF1, ARPC5L, SQLE, HMGCR, TBK1, COPS5, PSMC2, PSMD14, PDAP1, DDIT4, ADD3, DHCR7, SC5D, NMT1, HPRT1, SLC7A5, G6PD, SLC6A6, ENO1, ALDOA, TFRC, ITGB2, AK4, HMGCS1, UCHL5, PGM1, HSPA5, PSME3, ACTR3, ACSL3, PSMC6, PSMA4, GSR, INSIG1, PGK1, ATP2A2, CYB5B, GSK3B, SKAP2, NFYC, DAPP1, HSPA4, EIF2S2, YKT6, TRIB3, ACTR2, MTHFD2L, PPA1, PRDX1, EEF1E1, ELOVL5, SCD, ACLY, PSMB5
group 1	Healthy controls	
group 2	active AOSD	
Enrichment Score	0.62	
Net Enrichment Score	1.40	
nominal p-value	7.05E-03	
adjusted p-value	1.13E-02	
**GEO study ID**	**GSE112057**	NAMPT, RRM2, PLOD2, BUB1, NFIL3, CDC25A, SLC2A3, DAPP1, LTA4H, IDI1, PLK1, HK2, ELOVL5, UBE2D3, IFRD1, SKAP2, RIT1, ACTR2, BCAT1, ACSL3, SLC6A6, GBE1, SCD, MAP2K3, PSAT1, RAB1A, TCEA1, SLA, PDK1, ACTR3, HMGCR, TUBA4A, GLRX, PPP1R15A, GSK3B, POLR3G, MCM4, DHFR, CXCR4, TXNRD1, LDHA, LDLR, TBK1, AURKA, MTHFD2L, IDH1, HMBS, FGL2, TFRC, PGK1, SSR1, ATP6V1D, GAPDH, ADD3, BTG2, ETF1, ITGB2, MTHFD2, PSMC6, DDIT3, DHCR24, SERP1, HPRT1, EDEM1, NFYC, HSPA5, PSMD13, PSMD12, CCNG1, HMGCS1, PITPNB, GLA, AK4, INSIG1, PGM1, PSMA4, GSR, G6PD, MCM2, PHGDH, CDKN1A, SLC7A11, SC5D, EGLN3, IFI30, SQLE, PSME3, PSMA3, CD9
group 1	Healthy controls	
group 2	sJIA	
Enrichment Score	0.42	
Net Enrichment Score	1.70	
nominal p-value	4.93E-05	
adjusted p-value	1.58E-04	
**GEO study ID**	**GSE17590**	SLC2A3, LDHA, PLOD2, LTA4H, ACTR2, GAPDH, DDIT4, NFIL3, ENO1, ALDOA, IFI30, SLC7A5, ACTR3, SLA, ITGB2, GLRX, BCAT1, PGK1, CXCR4, PRDX1, MTHFD2, CORO1A, GCLC, SSR1, CANX, BUB1, SLC1A5, RPN1, ELOVL5, PGM1, PPA1, CDC25A, HK2, XBP1, MCM4, IDI1, ATP6V1D, HSP90B1, GPI, SLC2A1, TPI1, TBK1, G6PD, PPP1R15A, TFRC, UBE2D3, LDLR, CALR, M6PR, SCD, RAB1A, GLA, SERP1, ADD3, GBE1, NFYC, SLC9A3R1, CCNG1, ADIPOR2, FGL2, PHGDH, ACLY, COPS5, GSK3B, PSMD12, TES, MAP2K3, PSMC2, HMGCR, ARPC5L, DAPP1, PSMC6, RRM2, ETF1, PSMA3, EDEM1, SHMT2, PSMA4, GSR, IFRD1, MCM2, RIT1, GMPS, PITPNB, HPRT1, UCHL5, CD9, TXNRD1, IMMT, HSPD1, SDF2L1, PSMB5, INSIG1, CTSC, HSPE1, EBP, HSPA5, BTG2, SLC6A6, RDH11, PDK1, PSMD14, STIP1, TOMM40, PSPH, ATP2A2, CDKN1A, QDPR, UFM1, POLR3G, CTH, EEF1E1, CCNF, PSMD13, PSME3, PDAP1, PSAT1, TRIB3, PSMC4, DHCR7, P4HA1, RPA1, SQSTM1, TM7SF2, TCEA1, IDH1, PLK1, CYP51A1, NUP205, GOT1, CCT6A, HSPA4, SORD, GGA2, ME1, NFKBIB, TUBG1, MLLT11
group 1	Healthy controls	
group 2	sJIA pretreatment	
Enrichment Score	0.67	
Net Enrichment Score	1.95	
nominal p-value	1.00E-10	
adjusted p-value	3.20E-10	

Supplementary Table 3. Clinical information for bone marrow pathology studies.

Patients with MAS

Patient	Age (yr)	Diagnosis	Clinical description
1	14	sJIA and MAS	Recurrent fever, rash, arthritis, hypotension, hepatitis, carditis, pancreatitis, cytopenia, ferritin > 8000 ng/mL
2	43	AOSD and MAS	Recurrent fever, malaise, myalgia, rash, pancytopenia, ferritin > 60,000 ng/mL.
3	26	AOSD and MAS	recurrent fever, rash, arthritis, pulmonary hypertension, renal dysfunction, ferritin > 20,000 ng/ml. Patient succumbed to MAS.
4	2	viral MAS	EBV infection, fever, diarrhea, elevated AST/ALT, hepato-splenomegaly, anemia, leukopenia and ferritin 69,000 ng/mL.
5	22	viral MAS	EBV infection, fever, GI bleeding, liver failure, ferritin > 60,000 ng/mL. Patient died from multiorgan failure and septic shock.
6	38	viral MAS	EBV infection, history of Crohn's disease, rash, fever, pancytopenia, splenomegaly, and ferritin > 100,000 ng/mL. Patient died from intracranial hemorrhage.

Controls with normal bone marrow pathology

Patient	Age (yr)	Diagnosis	Clinical description
1	30	Anemia	Anemia and mild leukopenia of unclear etiology
2	43	hypogammaglobulinemia	Low IgG levels of unclear etiology. Family history of common variable immunodeficiency

Supplementary Table 4. Antibodies and sources.

Target Antigen	Conjugation	Clone	Company	Catalog #	Lot #	Dilution
Ly6C	PE/Cy7	HK1.4	Biolegend	128018	B295200	1:500
Ly6C	BV 605	HK1.4	Biolegend	128036	B156853	1:500
CX3CR1	PE	SA011F11	Biolegend	149006	B307671	1:500
CD11b	PB	M1/70	Biolegend	101224	B308096	1:500
Ly6G	APC	1A8	Biolegend	127614	B313371	1:500
Ly6G	FITC	1A8	Biolegend	127606	B277117	1:500
CD3e	FITC	145-2C11	Biolegend	100306	B215536	1:500
B220	FITC	RA3-6B2	Biolegend	103206	B230445	1:500
CD45.1	AF 647	A20	Biolegend	110720	B203773	1:500
CD45.2	PE/Cy7	104	Biolegend	109830	B161541	1:500
CD14	PE	M5E2	Biolegend	301850	B271173	1:500
CD14	PB	M5E2	Biolegend	301816	B257037	1:500
CD68	AF647	FA-11	Biolegend	137003	B253579	1:500
CD41	FITC	MWReg30	Biolegend	133904	B282876	1:500
TER-119	FITC	TER-119	Biolegend	116206	B208450	1:500
CD117 (c-kit)	PE/Cy7	2B8	Biolegend	105814	B205421	1:500
CD11b	FITC	M1/70	Biolegend	101206	B160103	1:500
CD16/32	PerCP/Cy5.5	93	Biolegend	101324	B197126	1:500
CD115	PE	AFS98	Biolegend	135505	B256877	1:500
CD34	PB	SA376A4	Biolegend	152204	B171705	1:500
CD4	PE/Cy7	GK1.5	Biolegend	100421	B312815	1:500
CD62L	FITC	MEL-14	Biolegend	104405	B258725	1:500
CD44	APC	IM7	ThermoFisher	48-0441-82	E08502-1631	1:500
Phospho-4EBP1 (T37/46)	AF647	236B4	Cell Signaling	5123S	9	1:100
Phospho-S6 (S240/244)	AF488	D68F8	Cell Signaling	5018S	6	1:100
Phospho-Akt (S473)	AF488	D9E	Cell Signaling	4071S	11	1:100
CD45R (B220)	FITC	RA3-6B2	ThermoFisher	13-0452-85	E02531-301	1:100
TSC2	unconjugated	D93F12	Cell Signaling	4308T	6	1:1000
β-actin	unconjugated	2F1-1	Biolegend	643802	B177370	1:2000
anti-mouse IgG	HRP	polyclonal	Cell Signaling	7076S	35	1:2500
anti-rabbit IgG	HRP	polyclonal	Cell Signaling	7074P2	30	1:2500

Supplementary Table 5. Gene sets and sources for gene set enrichment analysis.

Gene set	Source [9-11]
HEDGEHOG_SIGNALING	Hallmark gene sets
IFN-γ_RESPONSE	Hallmark gene sets
IL2/STAT5_SIGNALING	Hallmark gene sets
IL6/STAT3_SIGNALING	Hallmark gene sets
MTORC1_SIGNALING	Hallmark gene sets
MYC_TARGETS_V2	Hallmark gene sets
NOTCH_SIGNALING	Hallmark gene sets
TGFβ_SIGNALING	Hallmark gene sets
TNFA_SIGNALING_VIA_NFKB	Hallmark gene sets
UNFOLDED_PROTEIN_RESPONSE	Hallmark gene sets
WNT/β_SIGNALING	Hallmark gene sets
IL1_SIGNALING	Reactome Pathway
IL10_SIGNALING	Reactome Pathway
IL17_SIGNALING	Reactome Pathway
IL7_SIGNALING	Reactome Pathway
IL18_SIGNALING	WikiPathways

Supplementary Figure 1

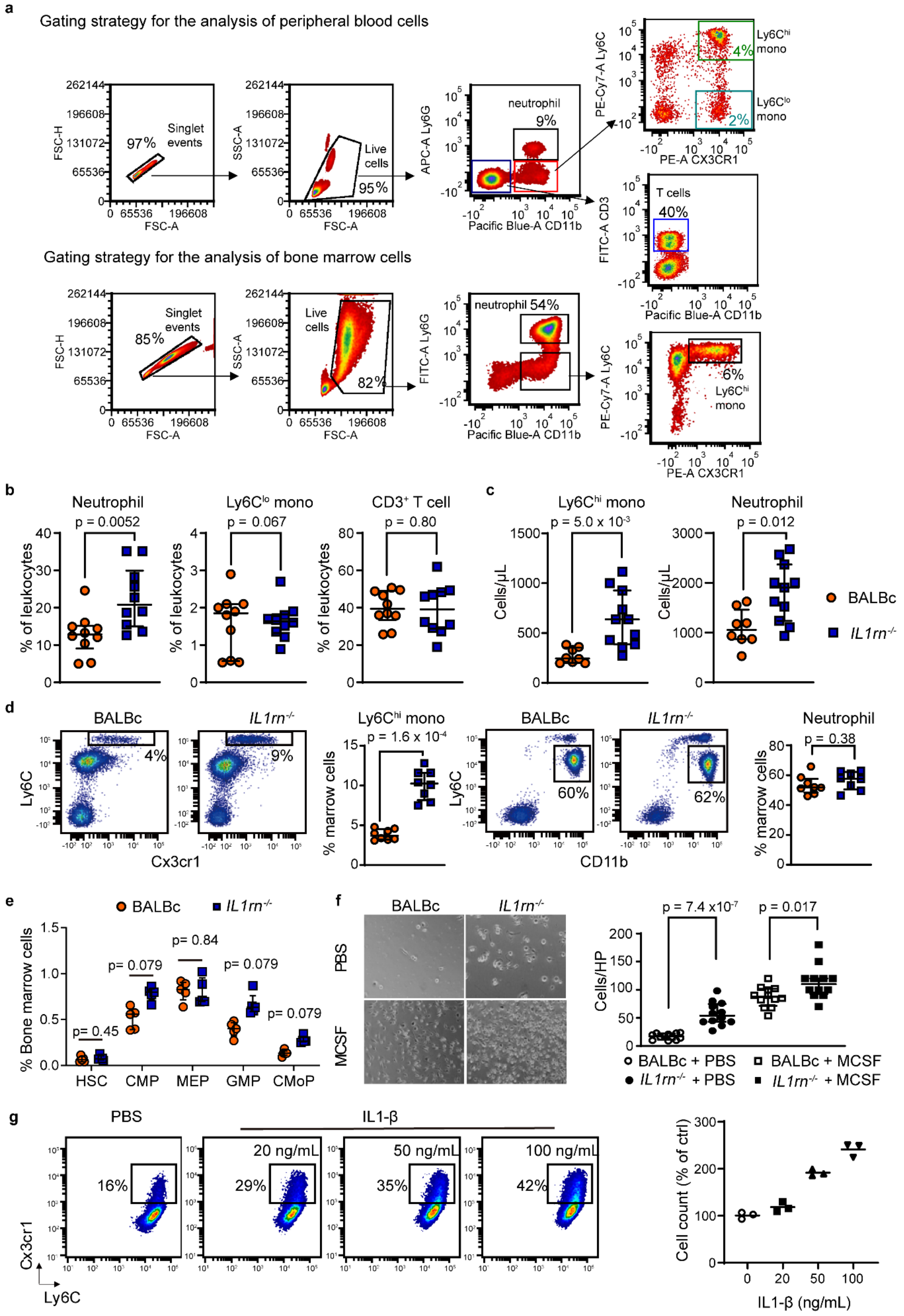

Supplementary Figure 1. Enhanced myeloid cell differentiation in IL1rn$^{-/-}$ mice. a) Gating strategy for flow cytometry analysis of peripheral blood cells and bone marrow cells. Percentages represent the percentage of gated events in live cells. b) Relative (n= 10 per group) and c) absolute count of cell subsets in peripheral blood of BALBc and IL1rn$^{-/-}$ mice (BALBc, n = 8, IL1rn$^{-/-}$ n = 11). d) Representative flow cytometry plot and quantification of bone marrow myeloid cell subsets in BALBc and IL1rn$^{-/-}$ mice (n = 8 per group). Boxes on left panels indicate Ly6C^{hi} monocytes and boxes on right panels indicate neutrophils. e) Quantification of hematopoietic stem cell (HSC), common myeloid progenitors (CMP), granulocyte-monocyte progenitors (GMP) and common monocyte progenitors (CMoP) in BALBc and IL1rn$^{-/-}$ mice (n = 5 per group). f) Representative photo (50x magnification) and quantification of adherent macrophages derived from cultured bone marrow cells in the presence or absence of M-CSF (3 high power fields at 100x magnification counted per sample; n = 4 mice per group). g) Expansion of ER-Hoxb8 myeloid progenitor cells in the presence of IL-1β (n = 3 per condition). Boxes indicate Ly6C^{hi} monocytes. Mice were 6-7 weeks old for all experiments and data in panels b-e were pooled from 2-3 independent experiments. Statistical analyses (all two-sided): Mann-Whitney U test (panels b-f). Median and error bars representing interquartile range are displayed in panels b-g Source data are provided as a Source Data file.

Supplementary Figure 2

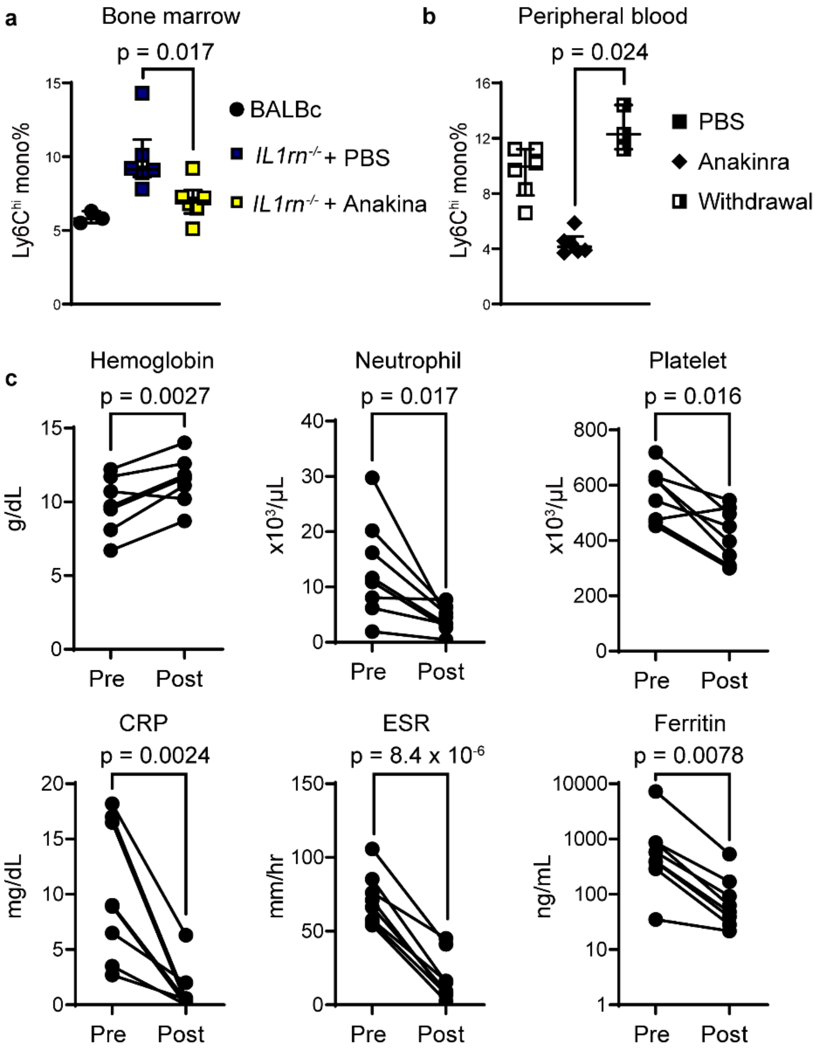

Supplementary Figure 2. Anakinra treatment reverses inflammation and monocyte expansion. a) Quantification of bone marrow Ly6C^{hi} monocytes in WT BALBc mice (n = 3) and IL1rn$^{-/-}$ mice treated with PBS (n = 6) or anakinra (n = 6; 10 mg/kg i.p. daily for 2 weeks). b) Quantification of peripheral blood Ly6C^{hi} monocytes in IL1rn$^{-/-}$ mice treated with PBS or anakinra for 2 weeks (n = 6 per group). Additional data were collected from the anakinra group 1 week after treatment discontinuation (n = 3). Mice were 8 weeks old for all experiments. Data in panels a and b were pooled from 2 independent experiments. c) Complete blood count and inflammatory marker measurements in sJIA patients pre-treatment and 2 weeks after initiation of anakinra (n = 8). Statistical analyses (all two-sided): Mann-Whitney U test (panel a,b); Wilcoxon signed-rank test (panel b). Median and error bars representing interquartile range are displayed in panels a,b. Source data are provided as a Source Data file.

Supplementary Figure 3

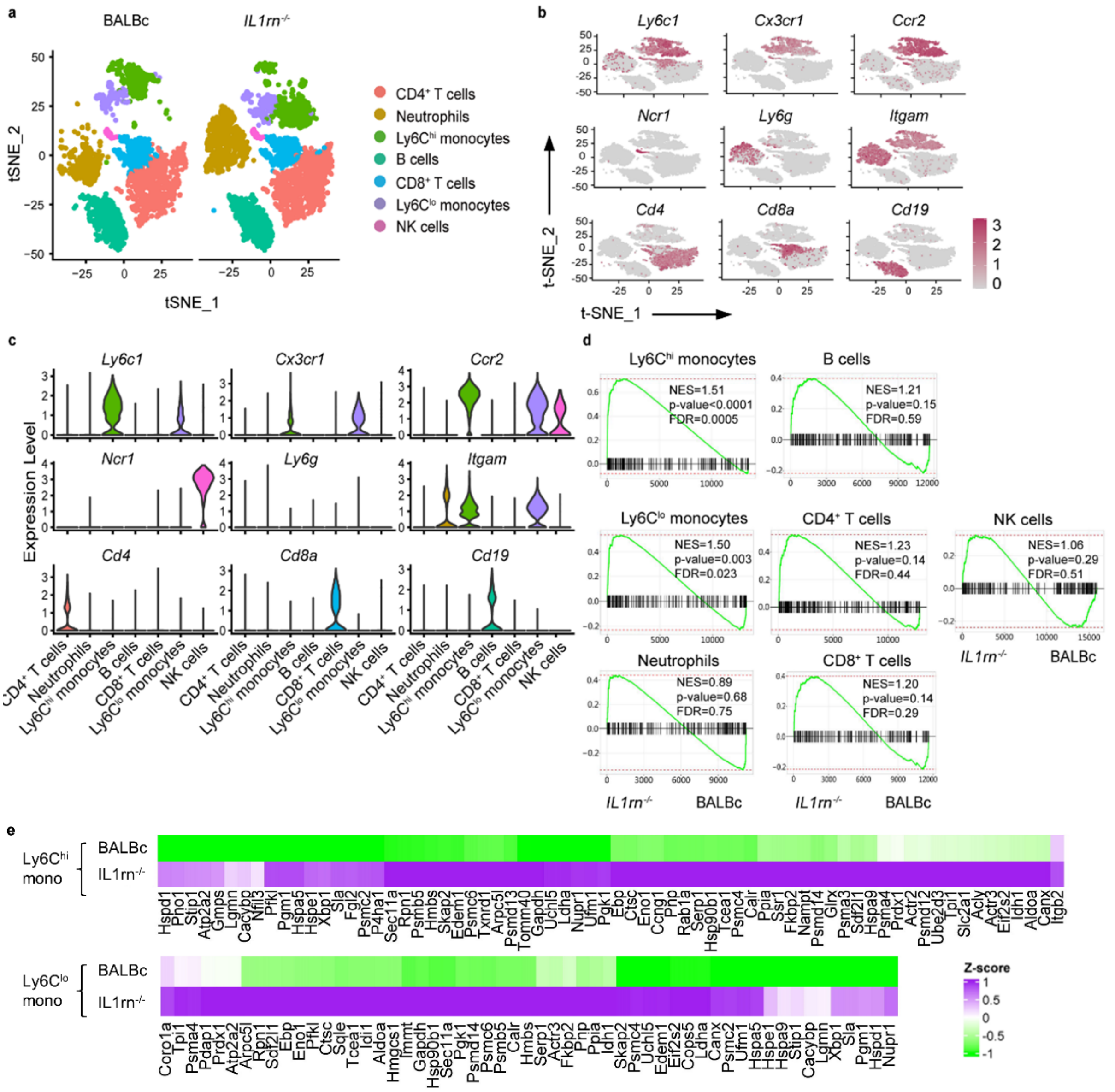

Supplementary Figure 3. Single Cell RNA-seq of peripheral leukocytes in IL1rn$^{-/-}$ mice. a) t-SNE plots of cell clustering based on scRNA-Seq of peripheral blood leukocytes from WT BALBc and IL1rn$^{-/-}$ mice (n = 4 pooled per group). Cells were classified into seven subclusters based on expression of lineage defining markers. b) t-SNE feature plots and c) violin plots illustrating the expression of lineage defining cellular markers. d) Gene set enrichment analysis of Hallmark mTORC1 gene set in peripheral blood cells from IL1rn$^{-/-}$ mice and WT BALB/c mice. e) Heatmap display of leading-edge genes from the mTORC1 gene set in Ly6C^{hi} monocytes and Ly6C^{lo} monocyte subsets of IL1rn$^{-/-}$ mice and BALB/c mice. Data present mean expression in specified monocyte populations. Mice were 8-9 weeks old for all experiments. Statistical analysis: permutation test (panel d). Source data are provided as a Source Data file.

Supplementary Figure 4

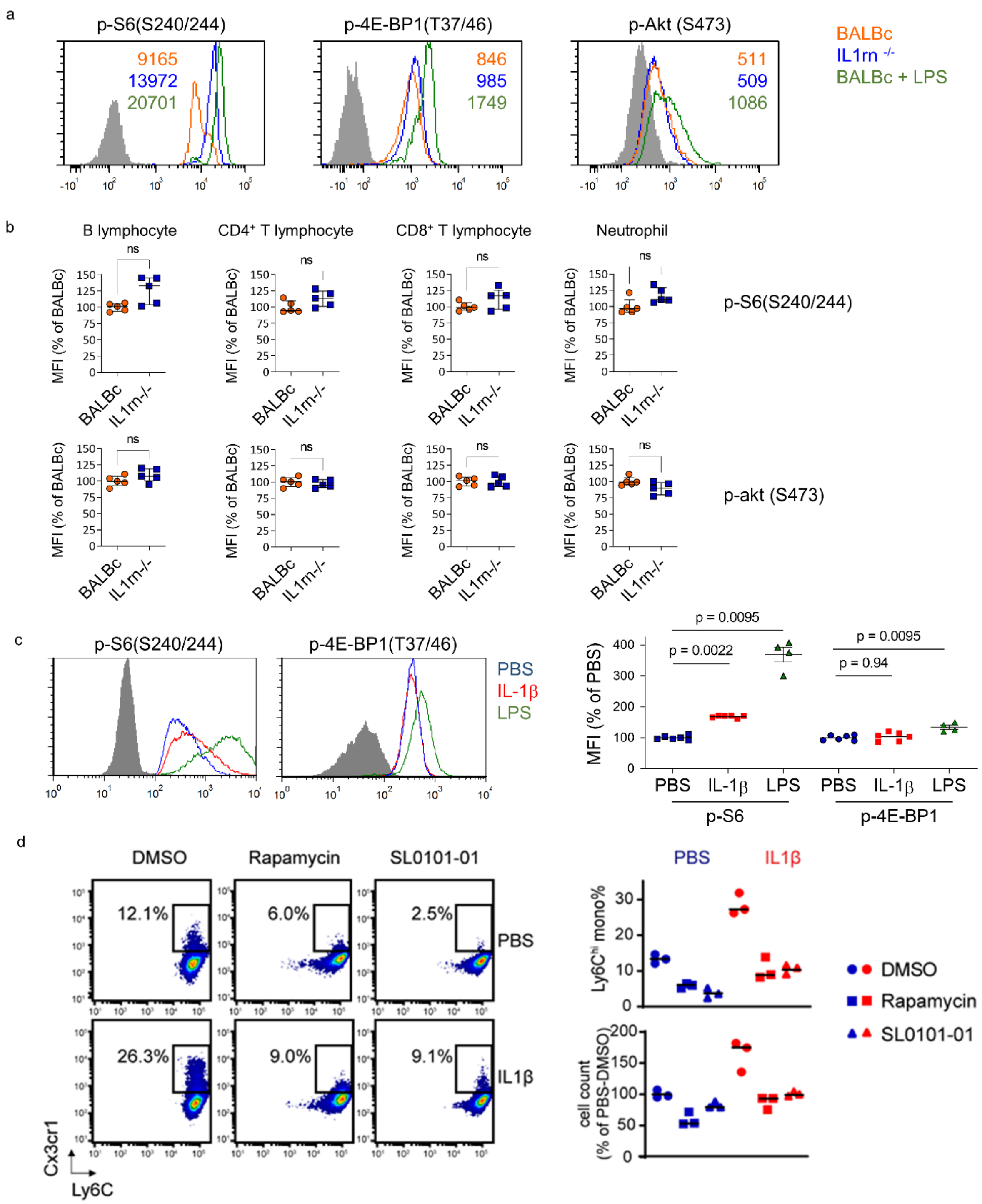

Supplementary Figure 4. mTORC1 mediates the inflammatory effects of IL-1 signaling. a) Phospho-flow analysis of mTOR substrates in bone marrow Ly6C^{hi} monocytes from WT and IL1rn$^{-/-}$ mice at baseline. Gray shades represent isotype control. Ex-vivo stimulation with lipopolysaccharide (100 ng/mL; 1 hr) was used as a positive control. b) phospho-flow analysis of S6 (S240/244) and Akt (S473) in bone marrow neutrophils and lymphocyte subsets from IL1rn$^{-/-}$ mice and WT BALB/c mice (n = 5 per group). c) Phospho-flow quantification of phosphorylated S6 (S240/244) and 4EBP1 (T37/46) in ER-Hoxb8 cells differentiated in the presence of IL-1β (50 ng/mL) or LPS (100 ng/mL). Mean fluorescence intensity was normalized to the mean of the PBS group. d) Flow cytometry plots and quantification of Ly6C^{hi} monocyte development from ER-Hoxb8 cells (box) treated with PBS or IL-1β, and in the presence of DMSO, rapamycin (500 nM) or SL0101-01 (10 nM; n = 3 per group). Mice were 8-9 weeks old for all experiments. Panels b and c are pooled from 2 independent experiments. Statistical analyses (all two-sided): Mann-Whitney U test (panels b and c). Median and error bars representing interquartile range are displayed in panels b-c. Source data are provided as a Source Data file.

Supplementary Figure 5

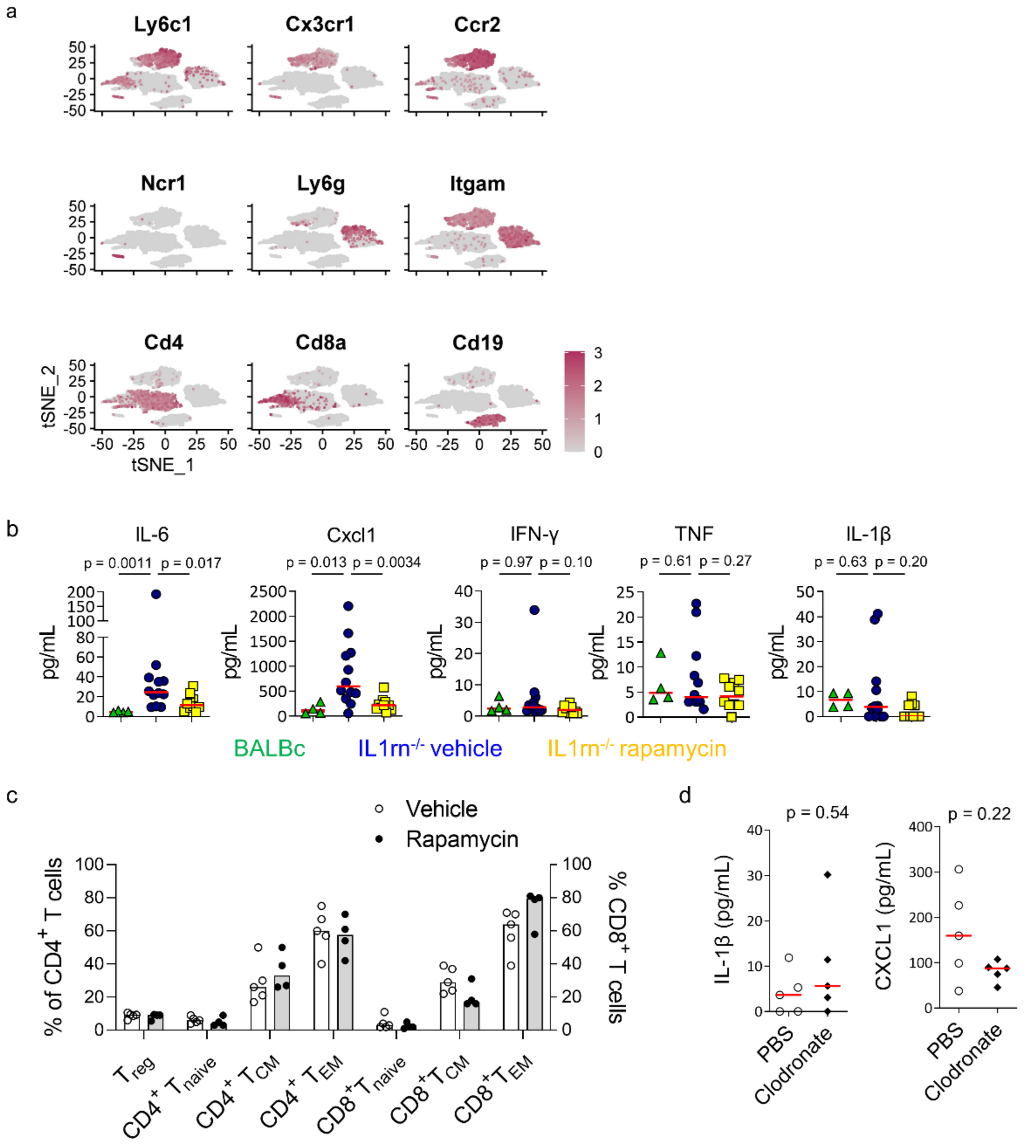

Supplementary Figure 5. mTOR inhibition and phagocyte depletion modulate the inflammatory signature in IL1rn$^{-/-}$ mice. a) t-SNE feature plots of single-cell RNA-seq data illustrating the expression of lineage defining cellular markers. b) Plasma cytokine / chemokine levels in BALBc mice and IL1rn$^{-/-}$ mice treated with rapamycin or vehicle control for 10 weeks (BALBc, n = 4; Vehicle, n = 12; Rapamycin, n = 10). c) Enumeration of naïve T cells (CD62L^{+} CD44^{lo}), central memory T cells (T_{CM}; CD62L^{+} CD44^{+}), effector memory T cells (T_{EM}; CD62L^{lo} CD44^{+}), and regulatory T cells (T_{reg}; CD4^{+} CD25^{+} Foxp3^{+}) in the spleen of IL1rn$^{-/-}$ mice treated with rapamycin or vehicle control (n = 5 per group). d) Quantification of plasma IL-1β and CXCL1 levels in mice treated with PBS-liposomes or clodronate-liposomes for 6 weeks (n = 5 per group). Mice were 8 weeks old for the experiment in panel a, 4 weeks old for the experiments in panels b, c, and 6 weeks old for experiments in panel d. Data in panels b were pooled from 2 independent experiments. Statistical analyses (all two-sided): Mann-Whitney U test (panels b, c and d). Median and error bars representing interquartile range are displayed in panel b. Bars in panel c and lines in panel d represent the median. Source data are provided as a Source Data file.

Supplementary Figure 6

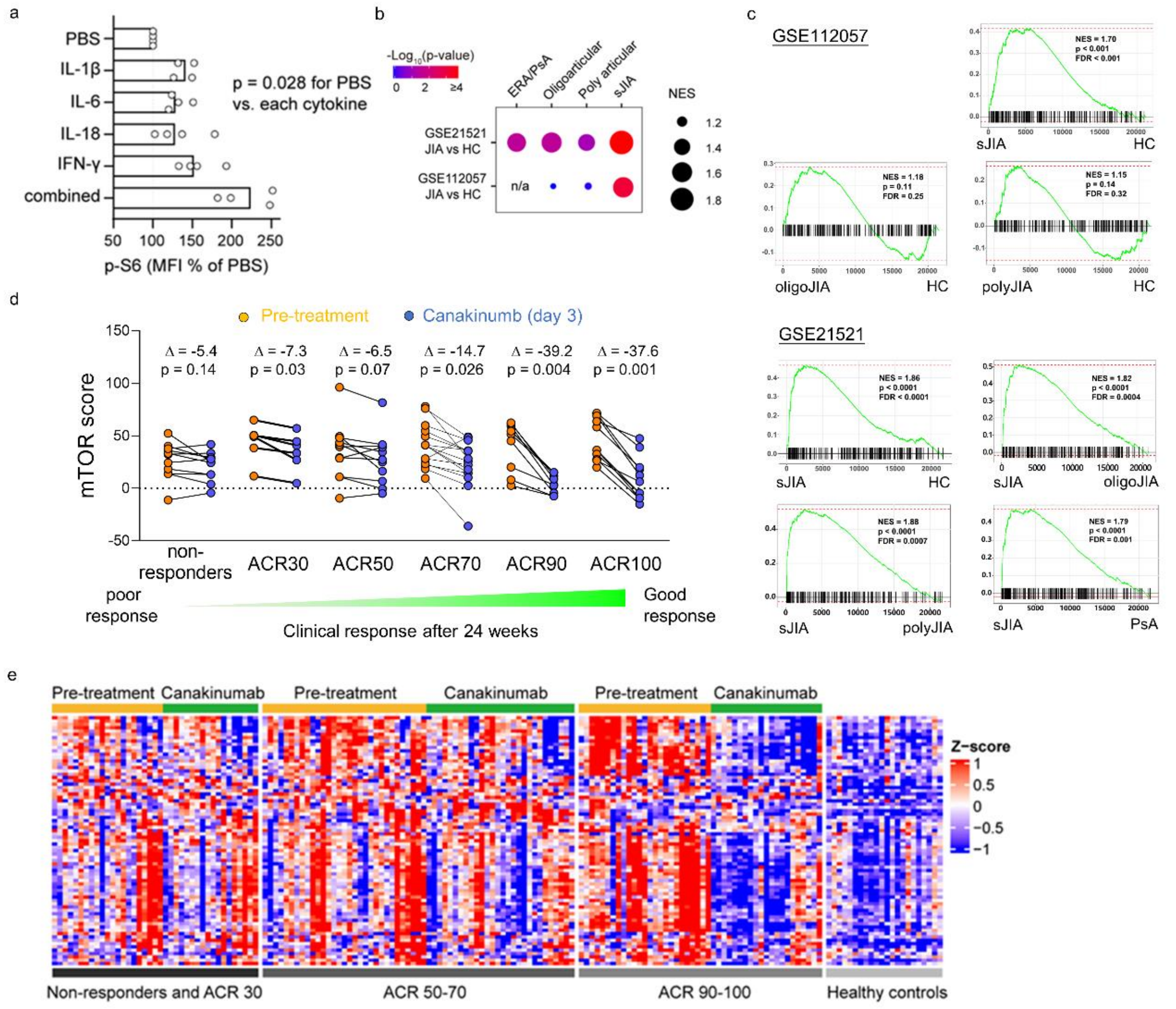

Supplementary Figure 6. mTORC1 transcriptomic signature in patients with SD. a) Phospho-flow quantitation of phosphorylated S6 (S240/244) and 4E-BP1 (T37/46) expression in human monocytes stimulated with proinflammatory cytokines individually or in combination. Data are normalized to MFI of PBS-treated cells. b) Cluster plot and c) GSEA plots of Hallmark mTORC1 gene set enrichment in patients with JIA subtypes relative to healthy controls (data source: GSE21521 and GSE112057). HC, healthy controls; PsA, psoriatic arthritis. d) Comparison of mTORC1 gene score changes after initiation of canakinumab (Day 3 minus baseline) and clinical response after 24 weeks (data source: GSE80060). e) Heatmap of leading-edge genes in the Hallmark mTORC1 gene set before treatment and 3 days after canakinumab treatment grouped by American College of Rheumatology clinical response after 24 weeks. weeks (data source: GSE80060). Statistical analyses (all two-sided): Mann-Whitney U test (panel a); permutation test (panel b,c), Wilcoxon signed-rank test (panel d). Bars in panel a represent the median. Source data are provided as a Source Data file.

Supplementary Figure 7

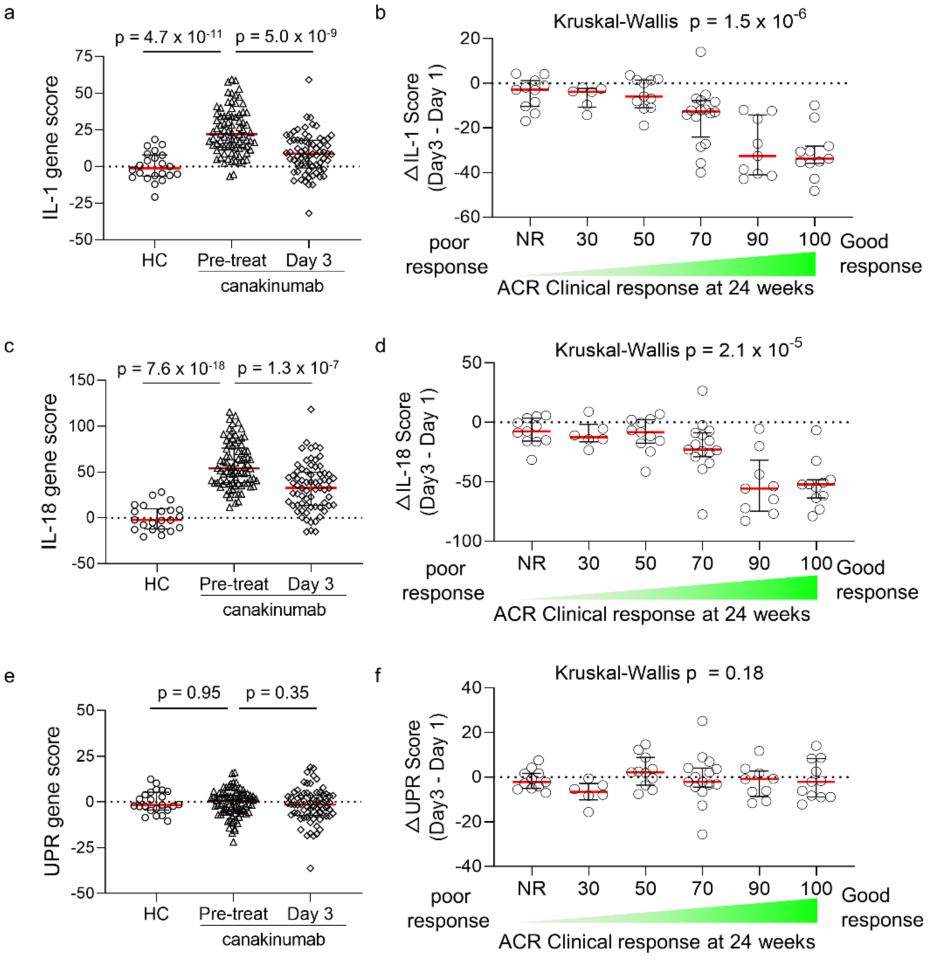

g

GSE80325	GSE17590	GSE21521	GSE80060	GSE147608	GSE7753	GSE113645	GSE112057	
↑	↑	↑	↑	↑	↑	↑		ALDOA
↑	↑	↑		↑	↑	↑		ENO1
↑	↑		↑				↑	GAPDH
↑	↑	↑			↑			GPI
↑	↑		↑				↑	HK2

Supplementary Figure 7. Transcriptomic and proteomic signatures of SD in published datasets. a-b) Expression of IL-1 signaling, (c-d) IL-18 signaling and (e-f) unfolded protein response gene sets in healthy controls (n = 22), sJIA patients at baseline (n = 82) and sJIA patients (n = 69) before treatment and 3 days after canakinumab treatment, and correlations with clinical response after 24 weeks (data source: GSE80060). Clinical response in b, d and f was stratified according to American College of Rheumatology (ACR) score: ACR 0 / Non-responders (NR, n = 10), ACR30 (n = 6), ACR50 (n = 11), ACR70 (n = 16), ACR90 (n = 8), ACR100 (n = 11). A standardized composite gene score for each pathway was calculated from the expression of leading-edge genes (comparing healthy controls and SD). g) Expression of genes encoding key glycolytic enzymes in patients with SD across different transcriptomic studies. Arrows indicate increased expression compared to controls from the same study ($p < 0.05$). Statistical analyses (all two-sided): Mann-Whitney U test (panels a, c, e and g); Kruskal-Wallace test (panel b, d and f). Median and error bars representing interquartile range are displayed in panels a-f. Source data are provided as a Source Data file.

Supplementary Figure 8

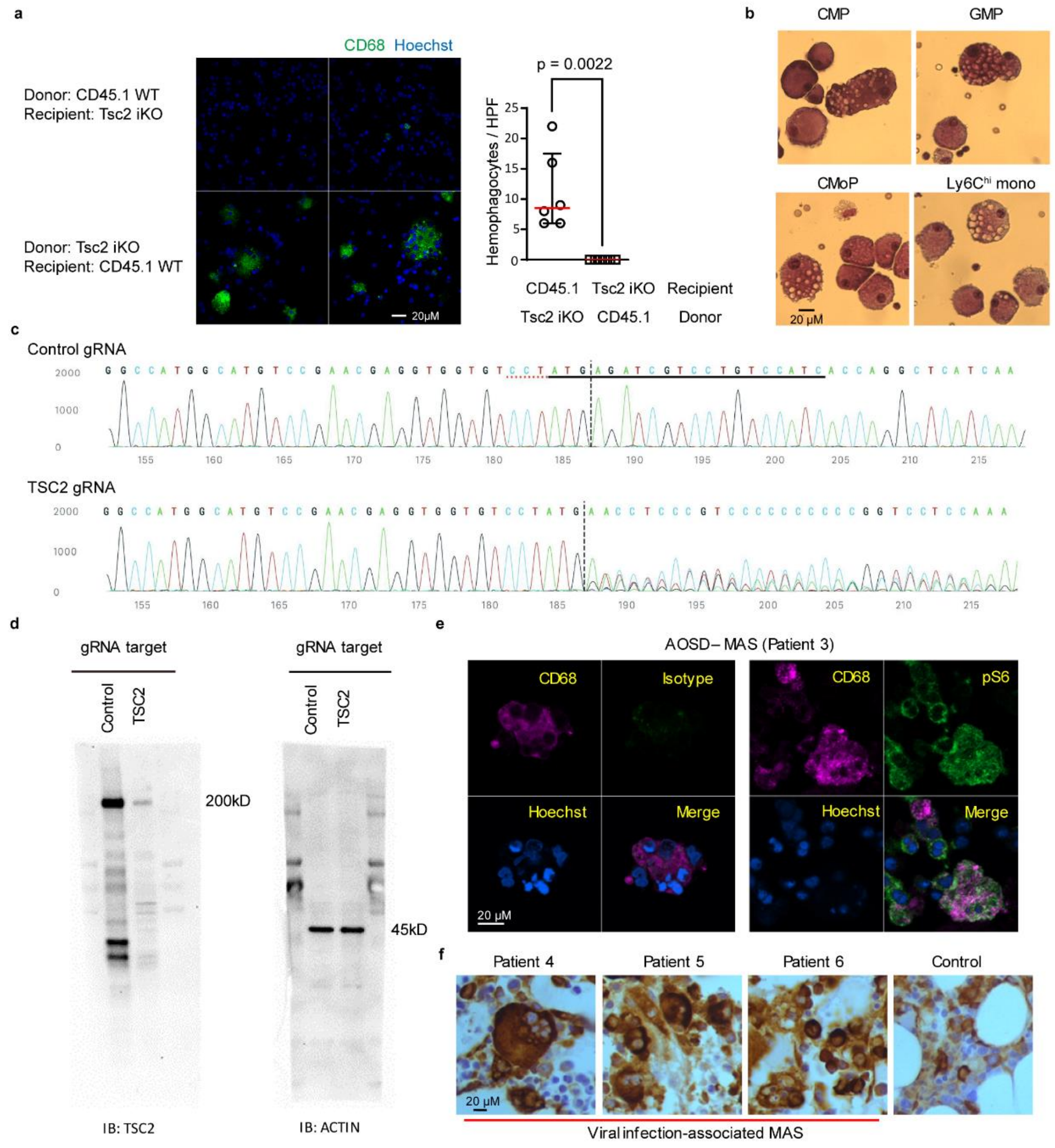

Supplementary Figure 8. Aberrant mTORC1 activation as a driver of hemophagocytosis. a) Representative confocal microscopy image and quantification of hemophagocytes in Tsc2 iKO mice transplanted with WT bone marrow and in WT mice transplanted with Tsc2 iKO bone marrow. Two high power fields (100x) were counted for each animal (n = 3 per group). Scalebar denotes 20 μM. Donor and recipient mice were 8 weeks old. b) Wright-Giemsa staining of sorted myeloid cell progenitors or monocytes from Ubiquitin-ERT2-Cre Tsc2 $^{fl/fl}$ mice cultured in the presence of 4OH-tamoxifen, SCF and M-CSF for 12 days and cocultured with erythrocytes for 24 hours. CMP, common myeloid progenitor; GMP, granulocyte-monocyte progenitor; CMoP, common monocyte progenitor. c) Sanger sequencing of monocytes electroporated with ribonuclear complex containing Cas9 and TSC2 guide RNA or control gRNA. Black line indicates gRNA sequence, red dotted line indicates protospacer adjacent motif and vertical black dotted line denotes predicted cleavage site with TSC2 gRNA. d) immunoblot of TSC2 and

beta-actin in human monocytes treated with TSC2-gRNA or control gRNA. Expected molecular weight for TSC2 (200 kD) and actin (45 kD) are indicated. Outside lanes were loaded with protein standards. e) Confocal microscopy of phospho S6 (S240/244) and isotype staining in hemophagocytes from a patient with fulminant AOSD. Scalebar denotes 20 µM. f) Immunohistochemistry of phospho-S6 (S240/244) in bone marrow section from patients with viral infection-associated MAS. Images in panels b, d are representative of 2 independent experiments. Images in panel e, f are derived from one experiment. Statistical analyses (all two-sided): Mann-Whitney U test (panel a). Median and error bars representing interquartile range are displayed in panel a. Source data are provided as a Source Data file.

Supplementary References

1 Cepika, A. M. *et al.* A multidimensional blood stimulation assay reveals immune alterations underlying systemic juvenile idiopathic arthritis. *J Exp Med* **214**, 3449-3466, doi:10.1084/jem.20170412 (2017).

2 Quartier, P. *et al.* A multicentre, randomised, double-blind, placebo-controlled trial with the interleukin-1 receptor antagonist anakinra in patients with systemic-onset juvenile idiopathic arthritis (ANAJIS trial). *Annals of the rheumatic diseases* **70**, 747-754, doi:10.1136/ard.2010.134254 (2011).

3 Ruperto, N. *et al.* Two randomized trials of canakinumab in systemic juvenile idiopathic arthritis. *The New England journal of medicine* **367**, 2396-2406, doi:10.1056/NEJMoa1205099 (2012).

4 Fall, N. *et al.* Gene expression profiling of peripheral blood from patients with untreated new-onset systemic juvenile idiopathic arthritis reveals molecular heterogeneity that may predict macrophage activation syndrome. *Arthritis and rheumatism* **56**, 3793-3804, doi:10.1002/art.22981 (2007).

5 Schulert, G. S. *et al.* Monocyte and bone marrow macrophage transcriptional phenotypes in systemic juvenile idiopathic arthritis reveal TRIM8 as a mediator of IFN-gamma hyper-responsiveness and risk for macrophage activation syndrome. *Annals of the rheumatic diseases*, doi:10.1136/annrheumdis-2020-217470 (2020).

6 Mo, A. *et al.* Disease-specific regulation of gene expression in a comparative analysis of juvenile idiopathic arthritis and inflammatory bowel disease. *Genome Med* **10**, 48, doi:10.1186/s13073-018-0558-x (2018).

7 Segawa, S. *et al.* Placenta Specific 8 Suppresses IL-18 Production through Regulation of Autophagy and Is Associated with Adult Still Disease. *J Immunol* **201**, 3534-3545, doi:10.4049/jimmunol.1800667 (2018).

8 Hinze, C. H. *et al.* Immature cell populations and an erythropoiesis gene-expression signature in systemic juvenile idiopathic arthritis: implications for pathogenesis. *Arthritis research & therapy* **12**, R123, doi:10.1186/ar3061 (2010).

9 Jassal, B. *et al.* The reactome pathway knowledgebase. *Nucleic Acids Res* **48**, D498-D503, doi:10.1093/nar/gkz1031 (2020).

10 Kelder, T. *et al.* Mining biological pathways using WikiPathways web services. *PLoS One* **4**, e6447, doi:10.1371/journal.pone.0006447 (2009).

11 Liberzon, A. *et al.* The Molecular Signatures Database (MSigDB) hallmark gene set collection. *Cell systems* **1**, 417-425, doi:10.1016/j.cels.2015.12.004 (2015).
