# Supplementary material for: mTORC1 links pathology in experimental models of Still’s disease and macrophage activation syndrome
Source: Nat Commun. 2022 Nov 28;13:6915. doi: 10.1038/s41467-022-34480-6 (PMC9705324; doi:10.1038/s41467-022-34480-6)
Supplement: Supplementary file 2 — Description of Additional Supplementary Files [file 41467_2022_34480_MOESM2_ESM.pdf]

Description of Additional Supplementary Files

Supplementary Data 1. Differential gene expression analysis of immune cell subsets from Single-cell RNAseq. Gene expression in B cells, $CD4^+$ T cells, $CD8^+$ T cells, NK cells, neutrophils, $Ly6C^{hi}$ monocytes and $Ly6C^{lo}$ monocytes were compared between BALB/c treated with vehicle vs. IL1rn $^{-/-}$ mice treated with vehicle, and between IL1rn $^{-/-}$ mice treated with vehicle vs. IL1rn $^{-/-}$ mice treated with rapamycin for 4 weeks (n = 4 pooled per group). Log2 fold change, nominal p-value and adjusted p-value are displayed for the comparison of each gene. Wilcoxon rank sum test with Bonferroni correction was used for statistical analysis.

Supplementary Data 2. Gene set enrichment analysis (GSEA) of single-cell RNAseq. GSEA analysis using the Hallmark collection was performed in B cells, $CD4^+$ T cells, $CD8^+$ T cells, NK cells, neutrophils, $Ly6C^{hi}$ monocytes and $Ly6C^{lo}$ monocytes from BALB/c treated with vehicle, IL1rn $^{-/-}$ mice treated with vehicle, and IL1rn $^{-/-}$ mice treated with rapamycin for 4 weeks (n = 4 pooled per group). The enrichment score, net enrichment score, nominal p-value, adjusted p-value, false discovery rate, gene set size, and leading-edge genes are listed for each gene set. Permutation test built-in the GSEA algorithm was used for statistical analysis. For the comparison of BALB/c and IL1rn $^{-/-}$ mice, a positive enrichment score denotes enrichment in IL1rn$^{-/-}$ group. For the comparison of IL1rn $^{-/-}$ mice treated with vehicle vs. rapamycin, a positive enrichment score denotes enrichment in rapamycin-treatment group.
