# Supplementary material for: mTORC1 links pathology in experimental models of Still’s disease and macrophage activation syndrome
Source: Nat Commun. 2022 Nov 28;13:6915. doi: 10.1038/s41467-022-34480-6 (PMC9705324; doi:10.1038/s41467-022-34480-6)
Supplement: Supplementary file 5 — Reporting Summary [file 41467_2022_34480_MOESM5_ESM.pdf]

nature portfolio

Corresponding author(s): Pui Lee and Peter A. Nigrovic

Last updated by author(s): Sep 12, 2022

Reporting Summary

Nature Portfolio wishes to improve the reproducibility of the work that we publish. This form provides structure for consistency and transparency in reporting. For further information on Nature Portfolio policies, see our Editorial Policies and the Editorial Policy Checklist.

Statistics

For all statistical analyses, confirm that the following items are present in the figure legend, table legend, main text, or Methods section.

n/a	Confirmed	
☐	☒	The exact sample size (n) for each experimental group/condition, given as a discrete number and unit of measurement
☐	☒	A statement on whether measurements were taken from distinct samples or whether the same sample was measured repeatedly
☐	☒	The statistical test(s) used AND whether they are one- or two-sided<br>*Only common tests should be described solely by name; describe more complex techniques in the Methods section.*
☒	☐	A description of all covariates tested
☐	☒	A description of any assumptions or corrections, such as tests of normality and adjustment for multiple comparisons
☐	☒	A full description of the statistical parameters including central tendency (e.g. means) or other basic estimates (e.g. regression coefficient) AND variation (e.g. standard deviation) or associated estimates of uncertainty (e.g. confidence intervals)
☐	☒	For null hypothesis testing, the test statistic (e.g. F, t, r) with confidence intervals, effect sizes, degrees of freedom and P value noted<br>*Give P values as exact values whenever suitable.*
☒	☐	For Bayesian analysis, information on the choice of priors and Markov chain Monte Carlo settings
☒	☐	For hierarchical and complex designs, identification of the appropriate level for tests and full reporting of outcomes
☒	☐	Estimates of effect sizes (e.g. Cohen's d, Pearson's r), indicating how they were calculated

Our web collection on statistics for biologists contains articles on many of the points above.

Software and code

Policy information about availability of computer code

Data collection	A description of the software and version for data collection has been included in the Methods.
Data analysis	A description of the software and version for data analysis has been included in the Methods.<br>Seurat package (v4.01). Code for Seurat is available at https://github.com/satijalab/seurat<br>limma package (v3.46.0). Code for limma is available at https://kasperdanielhansen.github.io/genbioconductor/html/limma.html<br>fGSEA package (v1.16.0). Code for fGSEA is available at https://github.com/ctlab/fgsea<br>complexheatmap package(v2.6.2). Code for complexheatmap is available at https://github.com/jokergoo/ComplexHeatmap<br>ggplot2 package (v3.3.3). Code for ggplot2 is available at https://github.com/z3tt/ggplot-courses<br>R (v4.0.4). R is available at https://cran.r-project.org/bin/windows/base/old/4.0.4/<br>GraphPad Prism (v.9.0).<br>BD FACS DIVA software (v.8.0)<br>FCS Express 5.0.

For manuscripts utilizing custom algorithms or software that are central to the research but not yet described in published literature, software must be made available to editors and reviewers. We strongly encourage code deposition in a community repository (e.g. GitHub). See the Nature Portfolio guidelines for submitting code & software for further information.

Data

Policy information about availability of data

All manuscripts must include a data availability statement. This statement should provide the following information, where applicable:

- Accession codes, unique identifiers, or web links for publicly available datasets
- A description of any restrictions on data availability
- For clinical datasets or third party data, please ensure that the statement adheres to our policy

Data Availability: Source data are provided with this paper. The Single cell RNA sequencing data used in this study are available in the NCBI Gene Expression Omnibus database under accession code GSE181243. Datasets analyzed in this study include GSE80325, GSE80060, GSE7753, GSE21521, GSE17590, GSE147608, GSE112057, and GSE113645.

Field-specific reporting

Please select the one below that is the best fit for your research. If you are not sure, read the appropriate sections before making your selection.

- [x] Life sciences
- [ ] Behavioural & social sciences
- [ ] Ecological, evolutionary & environmental sciences

For a reference copy of the document with all sections, see nature.com/documents/nr-reporting-summary-flat.pdf

Life sciences study design

All studies must disclose on these points even when the disclosure is negative.

Sample size	Sample sizes were not predetermined based on statistical methods. Sample sizes were determined based on preliminary experiments and historical data. n= 3 was chosen as the minimal replicate number for each condition.
Data exclusions	No data was excluded from the analyses.
Replication	With the exception of single cell RNA sequencing (with multiple animals pooled per group) and bone marrow immunohistochemistry studies on rare patient samples, all studies were independently replicated 2 to 3 times with consistent results.
Randomization	Mice were randomly assigned to treatment groups, and efforts were made to achieve equal representation of males and females in each experiment.
Blinding	Blinding was performed for measurement of arthritis and ankle erosion assessment. For other experiments, the investigators were not blinded during experiments and outcome assessment as the same investigators are involved in general animal husbandry and colony maintenance. Data analyses were performed independently by the principle investigators to minimize bias.

Reporting for specific materials, systems and methods

We require information from authors about some types of materials, experimental systems and methods used in many studies. Here, indicate whether each material, system or method listed is relevant to your study. If you are not sure if a list item applies to your research, read the appropriate section before selecting a response.

Materials & experimental systems

n/a	Involved in the study	
[ ]	[x]	Antibodies
[ ]	[x]	Eukaryotic cell lines
[x]	[ ]	Palaeontology and archaeology
[ ]	[x]	Animals and other organisms
[ ]	[x]	Human research participants
[x]	[ ]	Clinical data
[x]	[ ]	Dual use research of concern

Methods

n/a	Involved in the study	
[x]	[ ]	ChIP-seq
[ ]	[x]	Flow cytometry
[x]	[ ]	MRI-based neuroimaging

Antibodies

Antibodies used

Target Antigen,Conjugation,Clone,Company,Catalog #,Lot #,Dilution
Ly6C,PE/Cy7,HK1.4,Biolegend,128018,B295200,1:500
Ly6C,BV 605,HK1.4,Biolegend,128036,B156853,1:500
CX3CR1,PE,SA011F11,Biolegend,149006,B307671,1:500
CD11b,PB,M1/70,Biolegend,101224,B308096,1:500

Ly6G,APC,1A8,Biolegend,127614,B313371,1:500
Ly6G,FITC,1A8,Biolegend,127606,B277117,1:500
CD3e,FITC,145-2C11,Biolegend,100306,B215536,1:500
B220,FITC,RA3-6B2,Biolegend,103206,B230445,1:500
CD45.1,AF 647,A20,Biolegend,110720,B203773,1:500
CD45.2 ,PE/Cy7,104,Biolegend,109830,B161541,1:500
CD14,PE,M5E2,Biolegend,301850,B271173,1:500
CD14,PB,M5E2,Biolegend,301816,B257037,1:500
CD68,AF647,FA-11,Biolegend,137003,B253579,1:500
CD41,FITC,MWReg30,Biolegend,133904,B282876,1:500
TER-119 ,FITC,TER-119,Biolegend,116206,B208450,1:500
CD117 (c-kit),PE/Cy7,2B8,Biolegend,105814,B205421,1:500
CD11b,FITC,M1/70,Biolegend,101206,B160103,1:500
CD16/32,PerCP/Cy5.5,93,Biolegend,101324,B197126,1:500
CD115,PE,AFS98,Biolegend,135505,B256877,1:500
CD34,PB,SA376A4,Biolegend,152204,B171705,1:500
CD4,PE/Cy7,GK1.5,Biolegend,100421,B312815,1:500
CD62L,FITC,MEL-14,Biolegend,104405,B258725,1:500
CD44,APC,IM7,ThermoFisher,48-0441-82,E08502-1631,1:500
Phospho-4EBP1 (T37/46),Alexa Fluor 647,236B4,Cell Signaling,5123S,9,1:100
Phospho-S6 (S240/244),Alexa Fluor 488,D68F8,Cell Signaling,5018S,6,1:100
Phospho-Akt (S473),Alexa Fluor 488,D9E,Cell Signaling,4071S,11,1:100
CD45R (B220),FITC,RA3-6B2,ThermoFisher,13-0452-85,E02531-301,1:100
TSC2,unconjugated,D93F12,Cell Signaling,4308T,6,1:1000
β-actin,unconjugated,2F1-1,Biolegend,643802,B177370,1:2000
anti-mouse IgG ,HRP,Polyclonal,Cell Signaling,7076S,35,1:2500
anti-rabbit IgG ,HRP,Polyclonal,Cell Signaling,7074P2,30,1:2500

Validation

All antibodies have been validated by the following manufacturers Biolegend, Thermo Fisher and Cell Signaling Technology for flow cytometry and/or western blotting as indicated below. Details are directly obtained from the product websites.

Target Antigen,Clone,Company website,Verified Reactivity,Application

Ly6C,HK1.4,Biolegend,mouse,verified for flow cytometry
CX3CR1,SA011F11,Biolegend,mouse,verified for flow cytometry
CD11b,M1/70,Biolegend,"human, mouse, cynomolgus, rhesus",verified for flow cytometry
Ly6G,1A8,Biolegend,mouse,verified for flow cytometry
CD3e,145-2C11,Biolegend,mouse,verified for flow cytometry
B220,RA3-6B2,Biolegend,"human, mouse",verified for flow cytometry
CD45.1,A20,Biolegend,mouse,verified for flow cytometry
CD45.2 ,104,Biolegend,mouse,verified for flow cytometry
CD14,M5E2,Biolegend,"human, cynomolgus, rhesus",verified for flow cytometry
CD68,FA-11,Biolegend,mouse,verified for flow cytometry
CD41,MWReg30,Biolegend,mouse,verified for flow cytometry
TER-119 ,TER-119,Biolegend,mouse,verified for flow cytometry
CD117 (c-kit),2B8,Biolegend,mouse,verified for flow cytometry
CD16/32,93,Biolegend,mouse,verified for flow cytometry
CD115,AFS98,Biolegend,mouse,verified for flow cytometry
CD34,SA376A4,Biolegend,mouse,verified for flow cytometry
CD4,GK1.5,Biolegend,mouse,verified for flow cytometry
CD62L,MEL-14,Biolegend,mouse,verified for flow cytometry
CD44,IM7,ThermoFisher,"human, mouse",verified for flow cytometry
Phospho-4EBP1 (T37/46),236B4,Cell Signaling,"human, mouse, rat, hamster, monkey",verified for flow cytometry
Phospho-S6 (S240/244),D68F8,Cell Signaling,"human, mouse, rat, monkey",verified for flow cytometry
Phospho-Akt (S473),D9E,Cell Signaling,"human, mouse, rat, hamster, monkey, zebrafish, bovine",verified for flow cytometry
CD45R (B220),RA3-6B2,ThermoFisher,"human, mouse",verified for flow cytometry
TSC2,D93F12,Cell Signaling,"human, mouse, rat, monkey",verified for western blotting
β-actin,2F1-1,Biolegend,"mouse, human, rat",verified for western blotting
Horse anti-mouse IgG HRP-linked,Polyclonal,Cell Signaling,mouse,verified for western blotting
Goat anti-rabbit IgG HRP-linked,Polyclonal,Cell Signaling,rabbit,verified for western blotting

Eukaryotic cell lines

Policy information about cell lines

Cell line source(s)

ER–Hoxb8 myeloid progenitor (Mouse, Kindly provided by Dr. David B. Sykes)

Authentication

The authentication has been performed in the previous study. The study is available at https://immunology.sciencemag.org/content/2/11/eaam6641. Cells were authenticated based on morphology and ability to differentiate into monocytes and

	neutrophils after withdrawal of estrogen as described in Lee et al. Sci Immunol. 2017 May 26;2(11):eaam6641.
Mycoplasma contamination	Cell lines was tested for mycoplasma contamination and result were negative.
Commonly misidentified lines (See ICLAC register)	No commonly misidentified cell lines were used.

Animals and other organisms

Policy information about studies involving animals; ARRIVE guidelines recommended for reporting animal research

Laboratory animals	Wild-type BALB/c, wild-type C57BL/6, CD45.1 (B6.SJL-Ptprca Pepcb/BoyJ), ubiquitin ERT2-Cre (B6.Cg-Tg(UBC-cre/ERT2)1Ejb/1J), Mx1-Cre (B6.Cg-Tg(Mx1-cre)1Cgn/J) ,Tsc2 fl/fl (Tsc2tm1.1Mjg/J), and BALB/c Il1r1tm1Imx /J mice (IL1rn-/-) were used in the study. Male and female mice age between 4 to 10 weeks were used. Mice were subjected to a 12-hr light cycle (7A-7P). The relative humidity within the facility is kept at 50% and temperature is kept in the range of 22-23 degrees Celsius.
Wild animals	No wild animals were used in this study.
Field-collected samples	No field-collected samples were used in this study.
Ethics oversight	Animal studies were approved by the Institutional Animal Care and Use Committee (IACUC) at Brigham and Women's Hospital and Boston Children's Hospital.

Note that full information on the approval of the study protocol must also be provided in the manuscript.

Human research participants

Policy information about studies involving human research participants

Population characteristics	1) Children with newly diagnosed systemic juvenile idiopathic arthritis. 8 patients were recruited with median age of 5 years (4 males and 4 females)<br>2) adult and children diagnosed with macrophage activation syndrome and with bone marrow biopsy specimen available.
Recruitment	Recruitment was completed prior to the studies. All patients with sJIA from the recruitment period were included without additional selection.
Ethics oversight	Approval for human subject research and waiver of consent were granted by Institutional Review Board (IRB) of Massachusetts General Hospital (Protocol: 2017P000255) and Boston Children's Hospital (Protocol: P00005723).

Note that full information on the approval of the study protocol must also be provided in the manuscript.

Flow Cytometry

Plots

Confirm that:

- [x] The axis labels state the marker and fluorochrome used (e.g. CD4-FITC).
- [x] The axis scales are clearly visible. Include numbers along axes only for bottom left plot of group (a 'group' is an analysis of identical markers).
- [x] All plots are contour plots with outliers or pseudocolor plots.
- [x] A numerical value for number of cells or percentage (with statistics) is provided.

Methodology

Sample preparation	Whole blood from the mice was collected by the heart puncture or tail incision.<br>Bone marrow cells were obtained from the femurs. For phospho-flow cytometry, bone marrow cells were flushed from the femur with 4% paraformaldehyde.<br>Common myeloid progenitor , granulocyte-monocyte progenitors , common monocyte progenitor, and monocytes from from ubiquitin ERT2-Cre Tsc2 fl/fl mice were sorted by a FACSAriaTM Fusion Cell Sorter (BD).
Instrument	BD FACSCanto II™ Cell Analyzer equipped with 405 nm,488 nm and 633 nm lasers.<br>FACSAriaTM Fusion Cell Sorter (BD).
Software	BD FACSDiva Software (v.8.0. BD Biosciences) for data collection.<br>FCS Express 5 software (De Novo Software) for data analysis.
Cell population abundance	In the peripheral blood, Ly6C-high monocytes comprise 4-8%, neutrophil comprise 6-15%, and T cells comprise 35-45% of white blood cells. In the bone marrow, monocytes represent about 6-10% and neutrophils represent 50-60% of white blood cells.

Gating strategy

FSC-H and FSC-A channels were used to gate single cells from doublets. SSC-A versus FSC-A channels were applieds to separate the cells from debris. Gating strategy is provided in Supplemental Figure 1.

☒ Tick this box to confirm that a figure exemplifying the gating strategy is provided in the Supplementary Information.
